# Low-latency single channel real-time neural spike sorting system based on template matching

**DOI:** 10.1371/journal.pone.0225138

**Published:** 2019-11-22

**Authors:** Pan Ke Wang, Sio Hang Pun, Chang Hao Chen, Elizabeth A. McCullagh, Achim Klug, Anan Li, Mang I. Vai, Peng Un Mak, Tim C. Lei

**Affiliations:** 1 State Key Laboratory of Analog and Mixed-Signal VLSI, Institute of Microelectronics, University of Macau, Macau, China; 2 Department of Electrical and Computer Engineering, Faculty of Science and Technology, University of Macau, Macau, China; 3 Department of Physiology and Biophysics, University of Colorado Anschutz Medical Campus, Aurora, CO, United States of America; 4 Jiangsu Key Laboratory of Brain Disease and Bioinformation, Research Center for Biochemistry and Molecular Biology, Xuzhou Medical University, Xuzhou, China; 5 Department of Electrical Engineering, University of Colorado, Denver, CO, United States of America; Georgia State University, UNITED STATES

## Abstract

Recent technical advancements in neural engineering allow for precise recording and control of neural circuits simultaneously, opening up new opportunities for closed-loop neural control. In this work, a rapid spike sorting system was developed based on template matching to rapidly calculate instantaneous firing rates for each neuron in a multi-unit extracellular recording setting. Cluster templates were first generated by a desktop computer using a non-parameter spike sorting algorithm (Super-paramagnetic clustering) and then transferred to a field-programmable gate array digital circuit for rapid sorting through template matching. Two different matching techniques–Euclidean distance (ED) and correlational matching (CM)–were compared for the accuracy of sorting and the performance of calculating firing rates. The performance of the system was first verified using publicly available artificial data and was further confirmed with pre-recorded neural spikes from an anesthetized Mongolian gerbil. Real-time recording and sorting from an awake mouse were also conducted to confirm the system performance in a typical behavioral neuroscience experimental setting. Experimental results indicated that high sorting accuracies were achieved for both template-matching methods, but CM can better handle spikes with non-Gaussian spike distributions, making it more robust for *in vivo* recording. The technique was also compared to several other off-line spike sorting algorithms and the results indicated that the sorting accuracy is comparable but sorting time is significantly shorter than these other techniques. A low sorting latency of under 2 ms and a maximum spike sorting rate of 941 spikes/second have been achieved with our hybrid hardware/software system. The low sorting latency and fast sorting rate allow future system developments of neural circuit modulation through analyzing neural activities in real-time.

## Introduction

Recording action potentials from neurons in the brain gives neuroscientists the ability to study neural circuits with single cell accuracy [1–6] Typically neural spikes (or action potentials) are recorded *extracellularly* with a metal or glass electrode inserted into the brain of an animal or a human patient [7,8]. By contrast, intracellular or patch clamp recordings with glass pipettes are much less common *in vivo* because pulsation, movement of brain tissue and electrode contamination make them very challenging. Therefore, electrodes are typically placed within the extracellular space between neurons to capture neural spikes extracellularly. In this extracellular configuration, neural spikes generated from several adjacent neurons are often captured by the electrode at the same time, hereafter referred to as multi-units, making it challenging to determine the activity patterns of single neurons included in the recording. These multi-unit recordings are especially common when signals are measured from brain areas densely packed with neurons. For this reason, spike sorting algorithms are often used off-line to separate the neural spikes and assign them to different cluster groups [9–12]. The underlying principle of spike sorting relies on the fact that neural spikes originating from different neurons will have different temporal profiles. The temporal profiles of these neural spikes are dependent on the impedance of the extracellular fluid between the neurons and the electrode, the currents produced by each neuron, as well as the cell membrane area from which the ionic currents can reach the metal electrode [1,7,8].

There has been a sustained effort to develop better spike sorting algorithms aimed at increasing both the accuracy and the speed of the sorting process. From a mathematical perspective, spike sorting can be considered as an unsupervised classification problem, and several classification algorithms, including K-means, Expectation Maximization (EM) and Multivariate Gaussian Mixture, have been used to sort neural spikes [9,11]. Besides these classification algorithms, superparamagnetic clustering (SPC) was specifically designed for neural spike sorting [9]. SPC borrows the physical concept of magnetic thermal interaction and models neural spikes as magnetic spin elements. As the temperature rises, the neural spin elements fracture into distinct groups for spike classification. Aksenova et al. modeled neural spikes as self-oscillating nonlinear oscillators and can be expressed by trajectories in the phase space described by a perturbated ordinary differential equation [13,14]. Caro-Martin et al also extracted linear independent spike features based on shape, phase and distribution features for the spikes and sort neural spikes using the spike features based on a modified k-mean technique [15]. The advantage of using phase space features instead of temporal shapes to sort neural spikes is less prone to amplitude fluctuation and non-Gaussian distributed cluster structures. In addition, there are several other off-line spike sorting algorithms that the clustering is based on consensus-based modified k-mean techniques [16], variational Bayes [17,18], and maximum a posteriori [19] to improve sorting speed and accuracy.

In recent years, newer developments in spike sorting algorithms were focused on classifying a larger number of neural spikes measured from a neural probe with multiple recording sites or from an electrode array. Also, efforts were made to separate temporally overlapping neural spikes to improve sorting accuracy. Less sophisticated sorting algorithms typically reject these temporally overlapping neural spikes, while newer algorithms typically employ additional processing steps to handle neural spike duplication measured from multiple arrays and temporal spike overlapping within the same electrode. For spatial neural spike duplication, since neural spike currents emitted from a neuron reach the electrodes approximately at the same time or with a slight delay of no more than 1 ms [20], these newer spike sorting algorithms implement spatiotemporal masks to identify similar neural spikes in a nearby region arriving roughly at the same time and ascribed these duplicated spikes as the same spike [12,21]. Another approach is to choose the largest neural spike among all the measured signals as the representative spike for sorting [22]. Following these ideas, Masked-EM is an off-line neural spike sorting technique that can theoretically sort neural spikes recorded from a dense electrode with thousands of recording sites, and a mask was used to reduce the amount of neural data to be processed to the vincity of adjacent electrodes [12,23]. Jun et al. used a fast density-peak fitting method to rapidly process neural spikes recorded from high density probes [24], while Yger et al. also took a density approach and GPU parallelization to handle recording from thousands of electrodes [21]. For temporally overlapping neural spikes within the same electrode, the overlapping spikes can be temporally separated by matching the temporal shapes to a superimposed shape constructed from the non-overlapping neural spikes. Pachitariu et al. minimized the difference between the time-trace only containing the overlapping neural spikes to a superpositioned time-trace constructed from the non-overlapping spikes, and the firing times of spikes originating from different neurons were recovered from the overlapping spikes using this approach [20].

Despite the considerable progress towards achieving higher accuracy in spike sorting, almost all of these algorithms examine the entire recording based on an interative approach to optimize the sort. Therefore, these algorithms are fundamentally designed to sort pre-recorded neural spikes, making these algorithms difficult to be used in real-time to sort streaming neural spikes with almost immediate sorting outcomes, which is required for real-time closed-loop control. In addition, these algorithms commonly require a powerful computer equipped with multiple CPU or GPU cores to accelerate the calculation for the iterations, making these techniques difficult to be implemented with lightweight processing units for system miniaturization—but note that these algorithms are also designed to sort a large amount of neural spikes from multiple channels simultaneously.

Another approach is to use simpler and iterative-free algorithms to sort streaming neural spikes in real-time. These algorithms can typically be implemented using lighter weight processing electronics, such as a small integrated circuit (IC) chip, such that the electronics may be miniaturized for portable closed-loop neural controls in the future [25]. Closed-loop neural control is a neural control scheme in which the state of a brain or a neural circuit is determined by analyzing the measured neural signal or spikes, and intervening in the neural circuits based on the analyzed result in real-time. Under this closed-loop neural control scheme, low computational latency (i.e. less than 10 ms) to analyze the input neural data is desirable to allow an immediate feedback control [26,27]. This “closed-loop” approach is particularly important for experiments involving light-sensitive opsins, or optogenetic proteins, to manipulate neural circuits by optical illumination [28–30]. This new biochemical technique opens up new opportunities to manipulate neural circuits based on neural activity, and closed-loop neural control may be used in the future to manage neural disorders or to reduce side-effects during deep brain stimulation treatments [31]. Early attempts to develop an IC for neural recording were realized by Olsson and Wise [32]. In their design, the IC was capable of recording from multiple neurons and an efficient compression circuitry was implemented to allow transmission of a large amount of recorded data out of the IC for further analysis. Soon after, Chae et al. implemented a 128 channel neural recording IC with feature extraction to simplify the massive amount of collected data and to allow transmission through wireless communication without including spike sorting [33]. It wasn’t until Rutishauser used a high-performance computer system to realize spike sorting in real-time. With their efforts, neural spikes pre-recorded from the human medial temporal lobe were sorted using a software algorithm with satisfactory results, but the system remained large in size [34]. Integrating a spike sorting algorithm into an IC to reduce system size was first demonstrated by Karkare et al. in which a Euclidian distance based sorting algorithm was used [35,36]. Later, Gibson et al. developed an FPGA system in which the spike sorting algorithm used by Rutishauser was implemented, increasing the sorting speed 25 fold, with a worst-case latency of 11 ms [34,37]. Park et al. [38] designed a real-time spike sorting system based on Rutishauser with the abilities of online training and classification, their work also optimized memory usage during the template training phase. Franke et al. and Dragas et al. [39,40] employed a template matching filter with the advantage of doing the spike detection and classification at the same time, their work also had the capacity to classify overlapping spikes. Wouters et al. [41] proposed a similar template matching filter design as Franke, their system was optimized for threshold-based spike sorting system through suppressing interfering spikes. These template matching based spike sorting systems were verified to be effective but considerations of commonly seen non-Gaussian distributed spikes, such as burst firing and electrode drifting, is lacking. Despite all these efforts to create a real-time spike sorting system, some of the sorting algorithms used in these systems remain basic and more technical efforts are required to improve on sorting streaming neural spikes in real-time.

In the past, we developed a monolithic integrated circuit (IC) integrated with a low-noise high input impedance neural amplifier and a high current power source to simultaneously record neural activity and inhibit neural activity with optical illumination [8]. However, the IC has yet to be used for closed-loop neural control since it does not include spike sorting or processing units to analyze neural spikes to determine brain states in real-time. In order to fill this gap, this work builds upon our previous results to develop a low sorting latency and high throughput spike sorting unit on a field programmable gate array (FPGA), assisted by a desktop computer. Our FPGA has the capability to perform real-time spike sorting by matching cluster templates pre-calculated by the desktop computer with neural spikes collected during a short training period. In order to ensure proper template generation, the templates were generated by a desktop computer using a more sophisticated neural spike sorting algorithm—SPC [42]. The cluster templates were then transferred to the FPGA module for subsequent real-time spike sorting through matching the incoming neural spikes to these cluster templates [43]. In order to allow the FPGA to achieve optimal sorting accuracy under different noisy conditions, two template matching methods–Euclidean distance (ED) and correlational matching (CM)–were also implemented in the FPGA and the two methods are selectable by investigators to achieve optimal sorting accuracy according to the type of noise during experiments. Rigorous testing determined that ED yield slightly better sorting accuracy when the spikes are contaminated by Gaussian noise. CM, on the other hand, can handle spike amplitude fluctuations caused by the metal electrode slowly drifting away from its initial implanted position within the brain better, such as in long-term (minutes to hours) behavioral neuroscience studies performed on awake behaving animals. The algorithm was also compared with several off-line spike sorting algorithms indicating that the template matching technique achieves comparable sorting accuracies but has a three order-of-magnitude shorter sorting time.

With our approach, a maximal spike sorting rate of 941 spikes/second was achieved for a single electrode. This sorting rate is several times higher than the typical firing rates of neurons, preventing accidental loss of neural spikes in the sorting. The sorting latency of processing a neural spike was measured to be less than 2 ms, which should be fast enough to be used to analyze neural spike data in closed-loop neural control settings. The sorting rate and latency are approaching the theoretical limits set since the natural spike width of an action potential is ~1 ms, making the theortical maximum sorting rate ~1000 spikes/second for a single electrode. In addition, the FPGA can also handle a maximum of eight neural clusters, which is generally more than the number of neurons a middle to high impedance metal electrode can simultaneously record. The FPGA module implements all the necessary processing sub-units at the hardware level and does not rely on the assistance of the desktop computer once the cluster templates are transferred. The system was tested in a behavioral experiment in which neural spikes were recorded and sorted from the olfactory bulb of awake male C57BL/6 mouse in real-time, and the system was also compared to other off-line sorting algorithms with high sorting agreements.

## Methods

### System implementation

**Fig 1:**
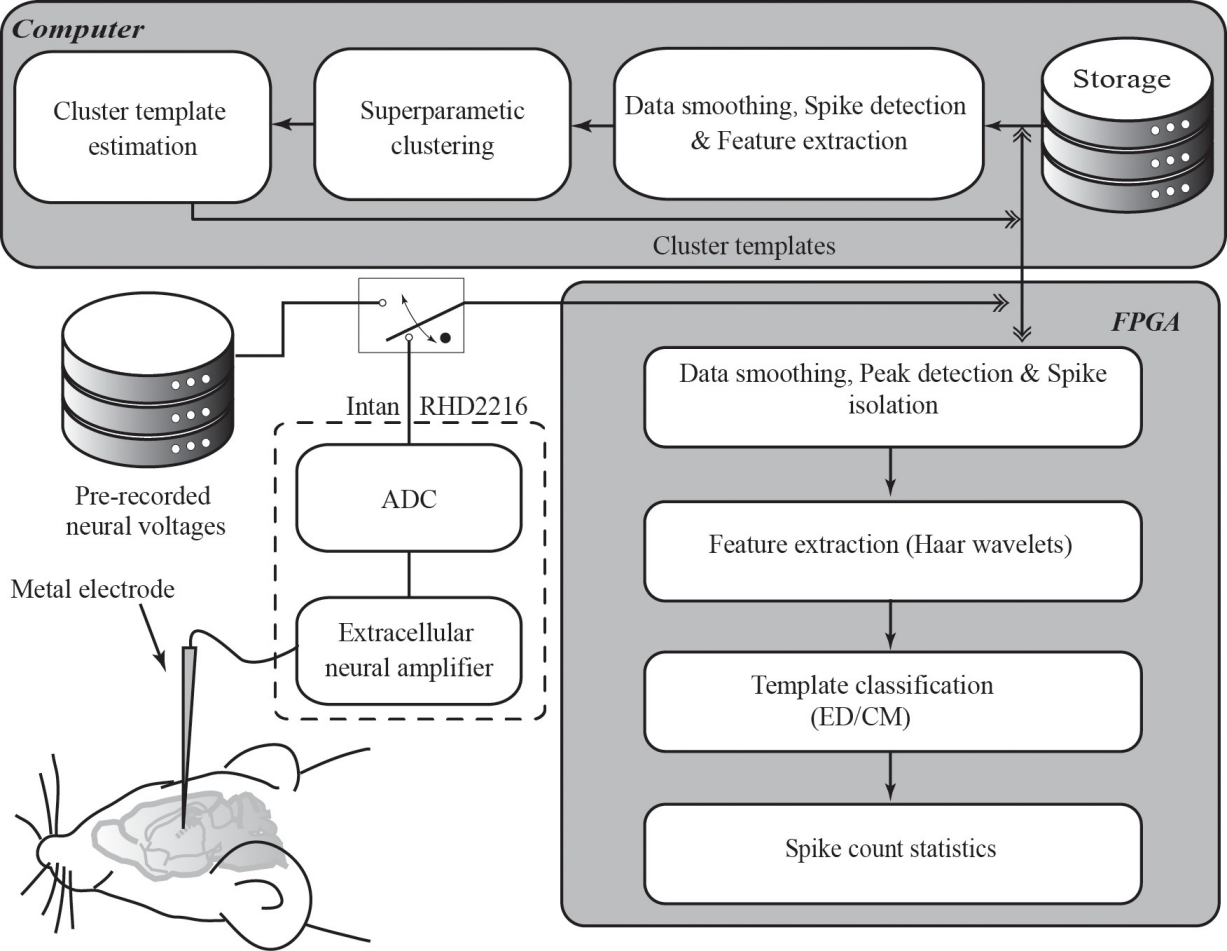
Block diagram of the real-time spike sorting system. The system is comprised of a desktop computer and an FPGA module. The system can measure extracellular neural spikes from an animal with a neural amplifier and an analog-to-digital converter (ADC), or alternatively be directly injected with digitized pre-recorded neural voltages for system testing. The desktop computer contains three sub-processing units– 1) raw data smoothing, spike detection and feature extraction, 2) spike sorting using SPC and 3) template estimation. The FPGA module also contains four sub-processing units– 1) raw data smoothing, peak detection and spike isolation, 2) feature extraction, 3) neural spike sorting based on template matching, and 4) calculation of spike count statistics.

The system was comprised of software and hardware components–a desktop computer running a spike sorting algorithm (software) to generate cluster templates during a short training period, and a FPGA module (hardware) to rapidly sort streaming neural spikes through template matching. Fig 1 is a schematic diagram illustrating the signaling between the desktop computer and the FPGA module, as well as the supporting electronic components for the desktop computer and the FPGA. In terms of functionality, the two components contain different processing sub-units to perform various tasks. The desktop computer has three software units to perform 1) raw data smoothing, spike detection and feature extraction, 2) spike sorting on the trained neural spikes using the SPC algorithm, and 3) template estimation based on the classification result. The FPGA module has four hardware units to perform 1) raw data smoothing, peak detection and spike isolation, 2) feature extraction, 3) streaming neural spike sorting based on template matching, and 4) calculation of spike count statistics based on the real-time sorting results. Here, units 1 and 2 were duplicated in the software and hardware systems to allow data comparison between real-time and off-line sorting. Despite the units being duplicated–one in software and one in hardware, only one was operating at a time. That is because once the cluster templates were estimated by the desktop computer and transferred to the FPGA, there was no need for the desktop computer to process the real-time data. The desktop computer, however, saved the real-time neural voltage trace, streaming from the FPGA, in its storage for performance evaluation.

For measuring neural spikes from the brain of a behaving mouse, an external low-noise amplifier (RHD2216, Intan Technologies, Los Angeles, CA) was used in front of the FPGA. The external amplifier has an internal band-pass filter for local field potential removal with a passband frequency from 300 to 5000 Hz. After the local field potential was filtered, an internal Analog to Digital Converter (ADC) digitized the analog neural voltage to an array of digitized voltage trace (12 bit) with a sampling frequency of 24 kHz, as shown in Fig 1. The digitized voltage trace was sent to the FPGA and passed to the desktop computer for cluster template estimation during the training period. In order to avoid excessive use of animals and to simplify the evaluation process, the external amplifier could be bypassed, and pre-recorded digitized neural spikes could be fed to the input of the FPGA in which all processing and calculation steps were identical to real-time animal recordings.

#### Desktop computer for cluster template estimation

The desktop computer contains a relatively powerful microprocessor (Intel Pentium i7), compared to the FPGA, and is capable of handling sophisticated spike sorting algorithms to allow more accurate estimation of cluster templates. Here we chose to use SPC as our spike sorting algorithm for cluster template generation. The advantage of SPC is that it does not require an estimation of the number of spike clusters contained in the digitized neural voltage x[n], as in the case for other simpler cluster algorithms (k-means), and is a well-accepted off-line spike sorting method in the neuroscience community. The desktop computer software contains three major processing sub-units– 1) spike detection and feature extraction, 2) SPC calculation, and 3) cluster template estimation–coordinating to generate accurate cluster templates for the FPGA.

**Spike detection and feature extraction in the phase space.** Digitized neural voltage x[n] measured during the training period was streamed from the FPGA to the desktop computer through a USB-UART port for cluster template estimation (details of measuring and converting the analog neural voltage to the digitized neural voltage is described in the FPGA hardware section). The digitized neural voltage x[n] can first be processed by an averaging filter to smoothen high frequency noise in the action potential signals (local field potential has been filtered before transferred to the computer, see below). In the literature, several signal enhancement methods have been used to determine the neural spike peak locations, including amplitude thresholding [42], nonlinear energy operator [33,35,44,45] and stationary wavelet transformation [46–48]. In our design, Nonlinear (or Teager) Energy Operator (NEO) was chosen to enhance the measured signal for better peak identification since NEO enhances both the instantaneous amplitude and the signal energy of the neural spikes [44]. NEO energy *x*_*NEO*_[*n*] was calculated based on the following equation.

$$x_{NEO}[n]=x{\left[n\right]}^{2}-x[n+1]∙x[n-1]$$ (1)

A threshold value *x*_*p*_, either three times the standard deviation of *x*_*NEO*_[*n*], or a value specified by investigators, was then used to compare to the calculated *x*_*NEO*_[*n*] for peak identification. After the peaks were identified, neural spikes were then isolated from the continuous neural voltage trace *x*[*n*] into isolated arrays *x*^*i*^[*n*], where i was the sequential index for each isolated neural spike, and *n* = 0 *to* 31 for the 32 data points centering against the spike peak center.

Phase space features can be extracted from the isolated neural spikes using wavelet transform (WT). In contrast to WT, principal component analysis (PCA) is a more commonly used method and has been used by many off-line spike sorting algorithms to extract features from neural spikes [46,49,50]. However, PCA needs to determine the principal components using the recorded neural spikes before it can be used to extract phase space features, which can increase the training time for the system. One alternative technique is to use a set of predetermined functions as the principal components (or basis functions). Compare to PCA, WT can extract features in real-time through a set of predetermined wavelet functions. However, it is important to note that the quality of the extracted features is highly dependent on the wavelet functions chosen. Therefore Haar wavelets were used in our design due to the robustness of the Haar wavelets in recovering features from noisy spikes [42]. The mathematical expressions of the Haar wavelets are listed in Eqs (2) and (3), where *m* is the scale level; *k* is the time translation; *l* is spike window length; and *φ*[*n*] is the Haar mother wavelet [51].

$$\Psi_{m,k}[n]=2^{\frac{m}{2}}\varphi [n-\frac{k}{2^{m-1}}]$$ (2)

$$\varphi [n]=\left\{ \begin{array}{cc} 1 & 0\leq n<\frac{l}{2}\\ -1 & \frac{l}{2}\leq n<l\\ 0 & otherwise \end{array}\right.$$ (3)

The features *w* in the phase space at scale level *m* can then be calculated using the following equation,

$${w[n]=2}^{\frac{m}{2}}*\sum_{k=-\infty }^{\infty }x[n]*\Psi_{m,k}{[2}^{m}n-k]$$ (4)

**Template estimation based on Superparamagnetic Clustering.** After the phase space features were extracted, SPC was employed to estimate cluster templates. Mathematically speaking, all spike sorting algorithms can be considered as unsupervised clustering methods in which neural spikes with similar features are assigned to the same cluster group. Therefore, many standard unsupervised clustering techniques, including K-means, K-means++ and Fuzzy c-means, have been used for spike sorting [33,46]. However, all of these clustering techniques require prior knowledge of the cluster number (the value K), which is often unknown during an experiment. The cluster number also depends on many experimental parameters, such as the relative position of the measuring metal electrode to the neurons. For this reason, nonparametric clustering algorithms were used in our design. SPC is an unsupervised spike sorting method that is well accepted by the neuroscience community for off-line spike sorting studies [9,42,49]. SPC has been demonstrated to improve sorting accuracy compared to other parametric sorting methods. An overview and comparison between the spike sorting techniques can be found in [49]. In short, the SPC algorithm was inspired by statistical mechanics in which phase transitions of micro magnetic domains occur as ambient temperature increases in a magnet. Based on this idea, the SPC algorithm randomly assigns the extracted features in the phase space of a neural spike with a spin value, and the spin values of all the spikes constitute a spin state of the entire recording. The total internal energy of a spin state can be calculated by summing the mutual interaction energies of all the spin states in which the mutual interaction energy is not zero and depends on the mutual distance only when the two spikes have different spin values. The probability distribution of the total internal energy of the spin state follows the Boltzmann distribution, as in a real physical magnetic system. Monte-Carlo techniques (Swendsen-Wang or Wolff techniques) are used to select a limited number of spin states to approximate the total probability distributions, and in turn, these spin states are used to estimate the clustering of micro-domains within the system [52–56]. The micro-domains tend to align uniformly at low temperature but align randomly at high temperature. This is due to the fact that lower energy states are more favorable at low temperature (ferromagnetic) and higher energy states are allowed as the temperature rises (paramagnetic). SPC occurs at a transition temperature between the ferromagnetic and paramagnetic states in which nearby neural spikes are clustered into a micro-domain. At this transition temperature, the center of these clusters can be considered as cluster templates to allow rapid matching to features of incoming neural spikes converted to the phase space.

The in-house software of the desktop computer was written in Python with the QT library for the user interface. The software then integrated the SPC algorithm to sort the neural spikes recorded during the training period to estimate for the cluster centers. After the cluster templates were estimated, the cluster templates were transferred to the FPGA module through the USB-UART port for real-time spike sorting during experiments.

#### FPGA module for sorting streaming neural spikes in real-time

After receiving the cluster templates expressed in the phase space from the desktop computer, streaming neural spikes can be sorted by the FPGA hardware in real-time through template matching. The FPGA module was implemented using an Arty FPGA development board (Xilinx, San Jose CA) which contains an Artix-7 35T FPGA chip. The FPGA was programmed using the Verilog hardware description language (HDL) with the Vivado Design Suite (Xilinx, San Jose, CA). Data communication between the desktop computer and the FPGA was through a 12 MBPS USB-UART board (FT4232H; Future Technology Devices International, Glasgow, Scotland). Once the real-time spike sorting began, the raw neural voltage trace was continually transferred from the FPGA to the desktop computer for storage purposes and the neural spikes were only processed by the FPGA during rapid sorting.

**Fig 2:**
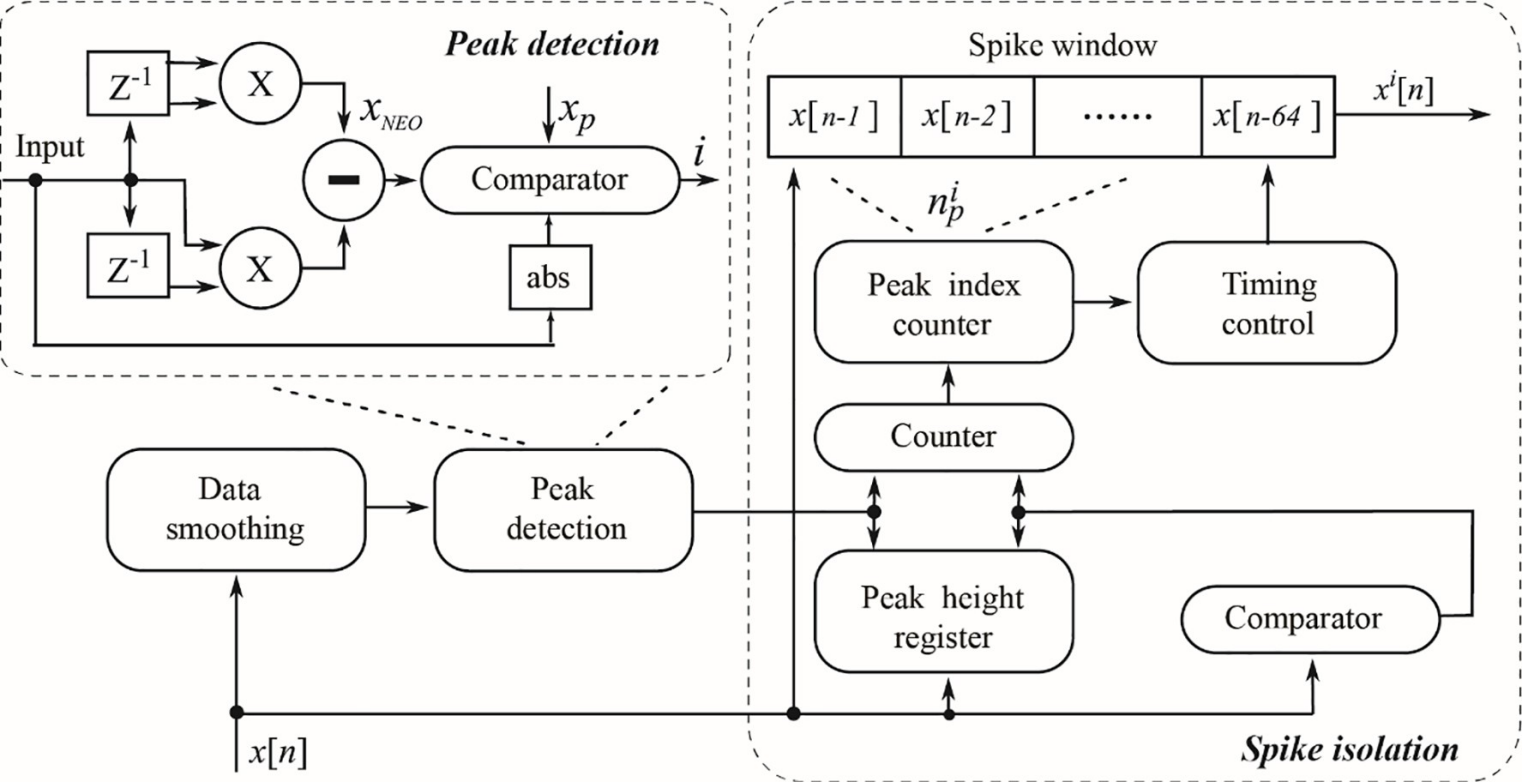
A block diagram illustrating the hardware implementation of the spike detection and isolation. An 8-sample smoothing filter was used to remove high frequency noise from the input neuron signal, followed by a peak detection module based on the NEO algorithm to detect a neural spike for isolation. A 64-sample FIFO was used to temporarily store the isolated data stream. A peak index counter and a peak height register worked synergistically to determine the peak index to correctly isolate the neural spike maximum. A 32-sample neural spike arrays centering against the spike peak center were outputted from the module for downstream feature extraction.

**Spike detection and Feature Extraction on the FPGA.** As shown in Fig 2, the same method used in the desktop computer software for spike detection, isolation and feature extraction were implemented on the hardware level, and only specific hardware implementation of these methods are described in this section to avoid duplication. Compared to the desktop computer software where the system memory is virtually unlimited, isolating a neural spike from the streaming neural voltage is much more challenging with hardware that has a very limited memory and computation capability. In our hardware implementation, an array of 32 data points was used to store the isolated neural spikes for subsequent data processing. However, simply isolating a neural spike based on the first peak crossing the threshold is problematic since noise contaminated neural spikes may have multiple peaks during which the highest peak should instead be chosen as the spike center. In order to correctly isolate a neural spike, 64 points of neural voltage data centered around the first peak position were temporarily stored in a piece of First-In-First-Out (FIFO) memory. A peak index counter was used to determine the offset value of the maximum peak height away from the first peak. A peak height comparator was then used to search for the maximum voltage after the first peak position and stored the offset to the peak index counter. Once the maximum peak was determined, the peak counter containing the offset value was used to reduce the 64 data points to 32 data points with the peak maximum is now placed at the center. The isolated neural spike array *x*^*i*^[*n*] was then sent to the hardware module implemented with a Haar wavelet transformation for feature extraction.

The Harr wavelet transformation module was hardware optimized for parallel computation to minimize the sorting latency. In the parallel design, the Haar transformer was divided into four levels and was implemented in the FPGA based on the following equations [57],

$$\begin{array}{l} \mathrm{Level}\;1\;d_{1}^{i}[n]=\frac{x^{i}[2n]-x^{i}[2n+1]}{\sqrt{2}}\\ \;a_{1}^{i}[n]=\frac{x^{i}[2n]+x^{i}[2n+1]}{\sqrt{2}}\\ \;\mathrm{for}\;n=0\ldots 15 \end{array}$$ (5)

$$\begin{array}{l} \mathrm{Level}\;2\;d_{2}^{i}[n]=\frac{a_{1}^{i}[2n]-a_{1}^{i}[2n+1]}{\sqrt{2}}\\ \;a_{2}^{i}[n]=\frac{a_{1}^{i}[2n]+a_{1}^{i}[2n+1]}{\sqrt{2}}\\ \;\mathrm{for}\;n=0\ldots 7 \end{array}$$ (6)

$$\begin{array}{l} \mathrm{Level}\;3\;d_{3}^{i}[n]=\frac{a_{2}^{i}[2n]-a_{2}^{i}[2n+1]}{\sqrt{2}}\\ \;a_{3}^{i}[n]=\frac{a_{2}^{i}[2n]+a_{2}^{i}[2n+1]}{\sqrt{2}}\\ \;\mathrm{for}\;n=0\ldots 3 \end{array}$$ (7)

$$\begin{array}{l} \mathrm{Level}\;4\;d_{4}^{i}[n]=\frac{a_{3}^{i}[2n]-a_{3}^{i}[2n+1]}{\sqrt{2}}\\ \;a_{4}^{i}[n]=\frac{a_{3}^{i}[2n]+a_{3}^{i}[2n+1]}{\sqrt{2}}\\ \;\mathrm{for}\;n=0\ldots 1 \end{array}$$ (8)

In this four level calculation, the Haar wavelet feature array ${\overset{\to }{w}}^{i}$ was constructed using the outputs in which ${\overset{\to }{w}}^{i}=\{a_{4}^{i},d_{4}^{i},d_{3}^{i},d_{2}^{i},d_{1}^{i}\}$. The hardware implementation details of the Haar wavelet transform were described in the S1 Text.

**Sorting streaming neural spikes using template matching.** The extracted features were then used to compare to the cluster templates in the phase space for neural spike classification. In our design, not all 32 wavelet features were used since significant classification information mostly gravitates towards the lower level features. Therefore, the template matcher was implemented to allow a maximum of 20 features to save system memory. Two template matching methods–ED and CM—were implemented in the FPGA for the classification of spikes. The method of sorting was determined by the users during the experiment. Generally speaking, ED gives a slightly higher sorting accuracy when the incoming neural spikes are mostly contaminated by Gaussian noise, and CM yields a better sorting accuracy for neural spikes mostly contaminated with non-Gaussian fluctuations (see results).

**Euclidean distance classifier.** The ED classifier implemented in the FPGA calculates the standard ED between the spike feature ${\overset{\to }{w}}^{i}$ to the eight cluster templates ${\overset{\to }{w_{t}}}^{a}$ (*a* = 1…8). The equation for the ED of the cluster templates ${\overset{\to }{w_{t}}}^{a}$ is

$$d_{a}^{i}=\sqrt{\sum_{n=0}^{31}{\left[(w^{i}[n]\right)}^{2}-{\left(w_{t}^{a}[n]\right)}^{2}]}$$ (9)

In the FPGA implementation comparing two ED values, a square operator ${\left(d_{a}^{i}\right)}^{2}$ was implemented instead of a square root operator, to reduce the complexity in constructing the comparators at the hardware level without scarifying accuracy.

**Correlational matching classifier.** A CM classifier was also implemented in the FPGA and was designed to handle up to eight cluster templates, and any unused correlators can be switched off if desired to save operational energy. Pearson’s correlation coefficient $\rho_{a}^{i}$ between the feature vector ${\overset{\to }{w}}^{i}$ of spike *i* with the cluster template ${\overset{\to }{w_{t}}}^{a}$ (*a* = 1…8) is defined as

$$\rho_{a}^{i}=\frac{{\overset{\to }{w}}^{i}*{\overset{\to }{w_{t}}}^{a}}{\sqrt{\left({\overset{\to }{w}}^{i}*{\overset{\to }{w}}^{i})({\overset{\to }{w_{t}}}^{a}*{\overset{\to }{w_{t}}}^{a}\right)}}$$ (10)

Where

$${\overset{\to }{w}}^{i}*{\overset{\to }{w_{t}}}^{a}=\frac{1}{32}\sum_{n=0}^{31}\left(w^{i}[n]-{\bar{w}}^{i})(w_{t}^{a}[n]-{\bar{w}}_{t}^{a}\right)$$ (11)

$${\overset{\to }{w}}^{i}*{\overset{\to }{w}}^{i}=\frac{1}{32}\sum_{n=0}^{31}\left(w^{i}[n]-{\bar{w}}^{i})(w^{i}[n]-{\bar{w}}^{i}\right)$$ (12)

$${\overset{\to }{w_{t}}}^{a}*{\overset{\to }{w_{t}}}^{a}=\frac{1}{32}\sum_{n=0}^{31}\left(w_{t}^{a}[n]-{\bar{w}}_{t}^{a})(w_{t}^{a}[n]-{\bar{w}}_{t}^{a}\right)$$ (13)

**Fig 3:**
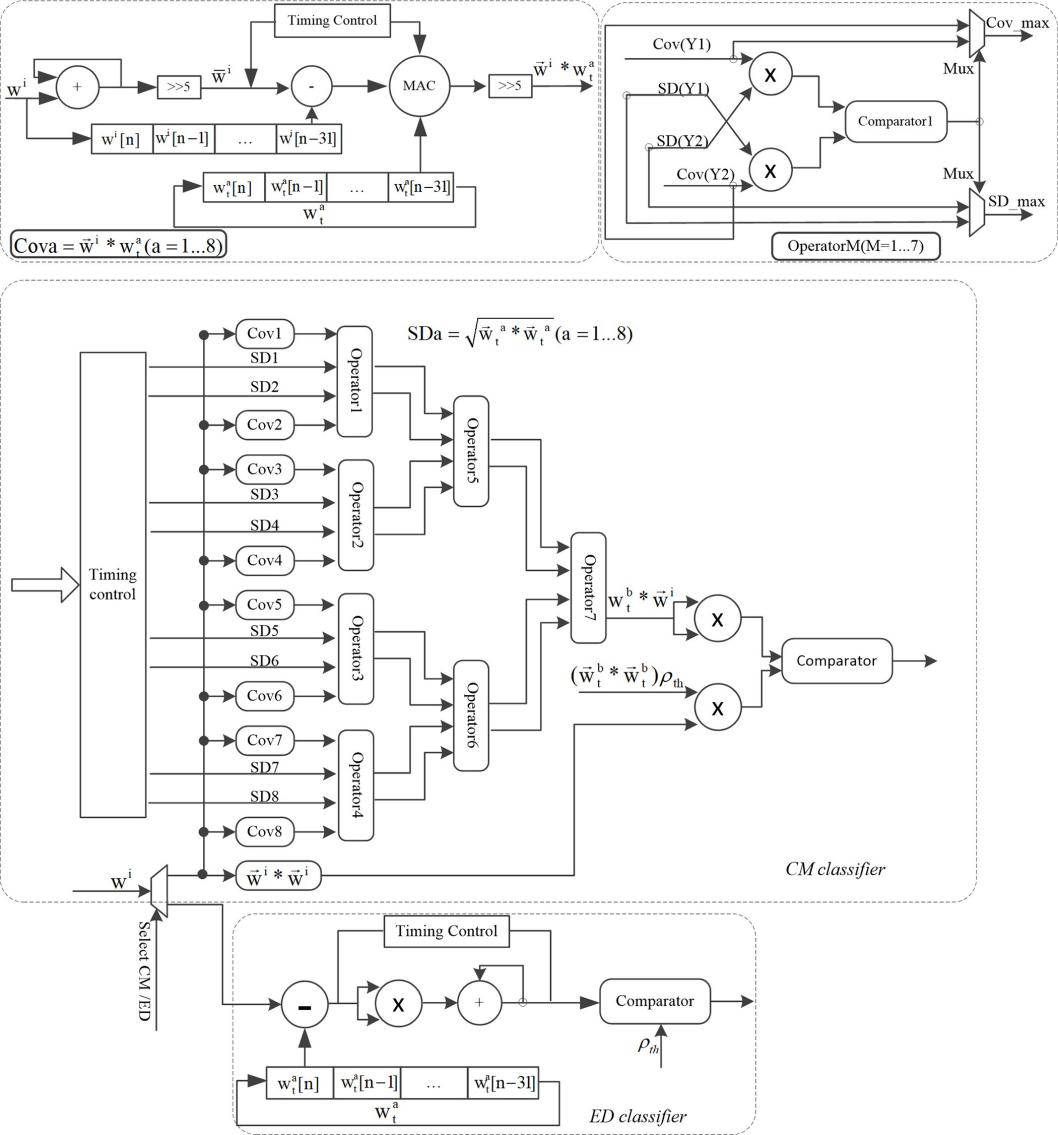
Hardware implementation of the CM and ED classifiers. Investigators can select one of the two classifiers through the “Select CM/ED” pin. Within the CM classifier, there are in total 8 covariance units (Cova) and 7 operator units (OperatorM) for determining the maximum correlation coefficient for the incoming spike to the eight cluster templates. Based on this design, the covariance calculations are performed in parallel to achieve minimum calculation latency. The hardware implementation of the covariance units, the operator units, and the ED classifier are also shown in detail on the top two sections and the bottom section of the figure.

${\bar{w}}^{i}=\frac{1}{32}\sum_{n=0}^{31}w^{i}[n]$ and ${\bar{w}}_{t}^{a}=\frac{1}{32}\sum_{n=0}^{31}w_{t}^{a}[n]$ are the averages of the wavelet features of the spike ${\overset{\to }{w}}^{i}$ and of the cluster template ${\overset{\to }{w_{t}}}^{a}$ respectively. As shown in Fig 3 and for each correlator, *w*^*i*^[*n*] was first summed together and subsequently right-shifted by 5 bits (equivalently divided by 2^5^ = 32) to calculate the average feature ${\bar{w}}^{i}$. *w*^*i*^[*n*] was also stored in a FIFO and then subtracted by the average ${\bar{w}}^{i}$ to calculate the difference $\left(w^{i}[n]-{\bar{w}}^{i}\right)$. Note that the two square-roots can be pre-calculated by the desktop computer using the cluster templates to reduce calculation burden for the FPGA hardware. The difference was then multiplied with the pre-calculated template difference $\left(w_{t}^{a}[n]-{\bar{w}}_{t}^{a}\right)$, summed together, and right-shifted by 5 bit to calculate the covariance ${\overset{\to }{w}}^{i}*{\overset{\to }{w_{t}}}^{a}$. In order to avoid calculating the square-root in the FPGA, the comparison between two correlation coefficients ($\rho_{a}^{i}$ and $\rho_{a\prime }^{i}$) was implemented with the expression below:

$$\left({\overset{\to }{w}}^{i}*{\overset{\to }{w_{t}}}^{a}\right)∙\left(\sqrt{{\overset{\to }{w_{t}}}^{a^{\prime }}*{\overset{\to }{w_{t}}}^{a^{\prime }}}\right)>\left({\overset{\to }{w}}^{i}*{\overset{\to }{w_{t}}}^{a^{\prime }}\right)∙\left(\sqrt{{\overset{\to }{w_{t}}}^{a}*{\overset{\to }{w_{t}}}^{a}}\right)$$ (14)

After three stages of comparison with eight comparators in total, the cluster template ${\overset{\to }{w}}_{t}^{b}$ best matching to the spike wavelet feature ${\overset{\to }{w}}^{i}$ was determined. In order to screen out abnormal spike shapes, for instance two very closely timed neural spikes that are overlapping and not well-matched to any of the cluster templates, a final comparator with a user-specified rejection threshold *ρ*_*th*_ was added at the end of the calculation pipeline to reject the outlier spikes that are not suitable to assign to any one of the eight clusters:

$${({\overset{\to }{w}}^{i}*{\overset{\to }{w}}_{t}^{b})}^{2}>\left({\overset{\to }{w}}^{i}*{\overset{\to }{w}}^{i}\right)\left({\overset{\to }{w}}_{t}^{b}*{\overset{\to }{w}}_{t}^{b}\right)\rho_{th}$$ (15)

All the covariance and operator units were implemented in a parallel structure to maximize speed and reduce sorting latency. Detailed hardware implementation of the covariance units, the operator units, and the ED classifier were shown by the sub-figures of Fig 3.

**Spike count statistic module.** A real-time statistics unit was implemented to perform statistical analysis at the final stage of the FPGA. The spike rates (spikes per second) of each cluster were calculated based on the output of the classifiers. Eight counters with programmable timers were added to count the spikes that were classified to one of the eight cluster groups. The calculated firing rates were also transferred to the desktop computer through the USB-UART port for real-time monitoring using the custom software.

### System assessment with published data and actual neural recording

The system was evaluated using both publicly available neural spike data, pre-recorded neural data that were obtained via extracellular recordings from an anesthetized Mongolian gerbil, and real-time *in vivo* recording in an awake and behaving mouse.

#### Public extracellular spike data

A publicly available extracellular neural recording dataset was used to evaluate our system and details about the dataset can be found in [42]. The neural spikes in the dataset were labeled and therefore can be used to compare to the sorting results obtained from our system. The dataset contains 23 sets of data with different degrees of signal-to-noise ratios. Particularly, there are 20 sets of data that were contaminated with Gaussian noise and 3 sets of data that were contaminated with non-Gaussian noise, which allows the use of ED and CM methods in the template matching to compare sorting accuracies under different noise conditions. The dataset was also used to compare the sorting accuracies and time between ED and CM to several off-line spike sorting algorithms.

#### Pre-recorded neural spikes of a gerbil

A previously recorded extracellular voltage trace measured with a high-impedance Tungsten metal electrode (WEPT33.0B10, MicroProbes, Gaithersburg, MD, USA) from the fifth nerve (trigeminal) within the brainstem of an anesthetized Mongolian gerbil (*Meriones unguiculatus*) was used to assess the system’s performance. All experimental procedures complied with all applicable laws and National Institutes of Health guidelines and were approved by the University of Colorado Institutional Animal Care and Use Committee (IACUC). The details of the experimental setup and the recording procedure were discussed in our previous publication and are not repeated here [8].

#### Real-time spike sorting with an awake behaving mouse

Two male C57BL/6 mice (8–16 weeks old) were used for the recording. The mice were purchased from the National Rodent Experimental Animal Seed Center (Shanghai, China) and were housed in the animal facility at the Xuzhou Medical University. All experiments were performed according to protocols approved by the Xuzhou Medical University Institutional Animal Care and Use Committee.

Fine wire electrodes were implanted into the olfactory bulb of the mouse for the recording. These fine wire electrodes were inserted 4.0 mm anterior to bregma and 1.0 mm lateral from the midline into the animal’s skull, and were driven to an average depth between 1.8 and 2.0 mm targeting the ventral mitral cell layer [58,59]. These electrodes were nichrome wires coated with polyide (single-wire diameter 0.0005” 12.7 μm, coating 1/4 hard PAC, item no. PF000591. RO-800, Sandvik, Stockholm Sweden). The output ends of the electrodes were jammed electronically to gold-plated holes of an electrode interface board (EIB-16, Neuralynx, Bozeman, MT) with gold connection pins (EIB Pins Large, Neuralynx), and a screw used as the signal ground was secured to the animal’s skull 1 mm posterior from the bregma and 1 mm from the midline. The electrodes, the ground screw, and the interface board were sealed and fixed to the animal’s skull using dental acrylic. A custom aluminum head plate was also attached to the animal’s skull using several stainless-steel screws and dental cement for securing the animal’s head to a stationary mount during recording experiments.

After the mice were fully recovered from surgery, the mice were transferred to an induction chamber and fixed to a stationary mount using the aluminum head plate on the animal’s head. The stationary mount prevented any movement of the head of the animal, which allows stable neural spike recording in real-time. The animals were also supported by an air-buffered Styrofoam sphere, allowing the mice to freely walk on top of the sphere. Real-time neural recordings were performed using the electrode interface board on one of the fine wire electrodes. The measured neural spikes were sent to the FPGA and the custom software on the desktop computer generated spike templates. After the spike templates were generated, the templates were transferred to the FPGA for spike clustering of subsequent incoming neural spikes in real-time. A supplemental video (S1 Video) recorded during the experiment shows the real-time process of spike sorting with neural spikes recorded from an awake behaving mouse.

## Results

In this section, experimental results based on a publicly available dataset [42], pre-recorded neural data from an anesthetized Mongolian gerbil, and neural spikes recorded from an awake behaving mouse and sorted in real-time were used to evaluate the performance of the system.

### Performance of the FPGA real-time module

**Fig 4:**
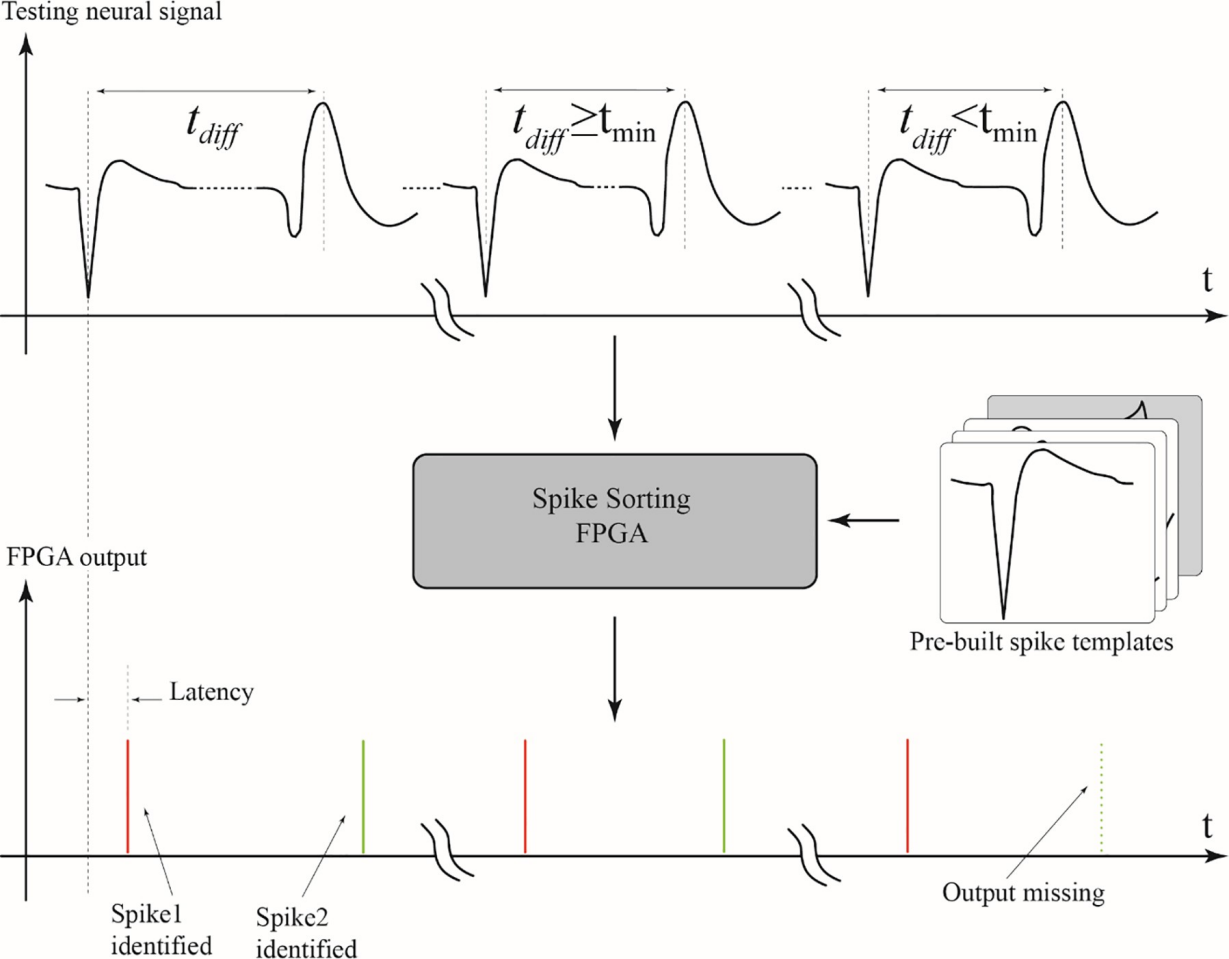
Estimate of maximum spike sorting rate and sorting latency for the system. Two neural spikes extracted from the neural recording of a gerbil were pieced together with a time difference *t*_*diff*_ to create an artificial voltage trace, which was sent to the FPGA hardware to estimate the maximum spike sorting rate. As the time difference *t*_*diff*_ between two spikes was monotonically reduced to *t*_*min*_, the FPGA hardware could no longer separate the two neural spikes and the voltage trace was considered as a single spike, resulting in missing classification for the second spike. The spike sorting latency of the system was also estimated by measuring between the time when the neural spike entered the FPGA for sorting and the time the FPGA resulted in a classification label for the neural spike.

The maximum spike sorting rate was first measured to characterize the FPGA performance. The maximum spike sorting rate was measured by monotonically reducing the temporal difference between the peaks of two spikes until the FPGA module can no longer differentiate the second spike from the first spike. Two neural spikes each with a data length of 32 time bins were selected from the previously recorded gerbil data and the two spikes were pieced together with a time difference *t*_*diff*_, as shown in Fig 4. If the temporal spacing between the two spikes was larger than the 32 time bins of the spikes, additional data points with no spike features were padded in the gap space, and if the spacing was less than the span, the data points of the overlapping space were averaged between the two spikes. The FPGA module was implemented with a digital output to indicate the successful sorting of the input spikes, as illustrated in Fig 4. Since the neural data was measured with a sampling rate of 24 kHz and if the two neural spikes were connected together back-to-back with no temporal padding, i.e. *t*_*diff*_ = 1.33 ms, a 750 spikes/second sorting rate will be obtained (Note that the spike itself is less than 32 time bins). The system can actually handle neural spikes more closely spaced together, making the maximum spike sorting rate higher than this number. In order to estimate the maximum sorting rate, the time difference between the two spikes was further reduced to allow overlaps, and the measured results indicated that the time difference between the two pulses could be as short as 1.06 ms (*t*_*min*_ = 1.06 ms). Thus, the maximum spike sorting rate of the FPGA module was determined to be 941 spikes/second.

The sorting latency of analyzing a neural spike for the FPGA module was also estimated and the data processing time for each of the sub-processing units are listed in Table 1. The sorting latency is the time required to sort a neural spike by the system from the time to start measuring the neural spike to the time delivering a sorting result. Comparatively, the sampling frequency (using an external analog-to-digital converter) in digitizing the neural voltage is 24 KHz, or 41.7 μs/sample, while the FPGA system clock frequency is 100 MHz, or 0.01 μs/clock, for data processing and calculations. Since the FPGA clock frequency is significantly higher than the sampling frequency, most of the latency resulted from the waiting time to collect enough data points to perform the sorting. Here the estimation of the sorting latency is briefly described. For the latency of data smoothing, an 8-point average moving filter was used in which the filter was required to wait for 4 additional digitized samples to be loaded into the filter before it could perform the smoothing calculation of the current data point. In addition, 2 additional FPGA processing clock cycles were required to calculate the average. Thus, the total processing time for the smoothing was 166.7 μs. For peak detection, the NEO algorithm was required to wait for 1 additional sample and 2 FPGA processing cycles for the calculation, equivalent to a latency of 41.7 μs. The spike isolation module was most time-consuming and thus was the dominant contributor to the sorting latency besides signal sampling. The module needs 32 sampling cycles to store the entire spike to its FIFO and another 10 additional samples to allow alignment of spikes with uneven spike shapes to the array center, and also required 32 FPGA processing clock cycles for the spike readout, which translates to a latency of 1750.3 μs. After the neural spike was isolated to 32 time bins, no additional data waiting was needed for the processes of Haar transformation, template matching and firing rate calculation, resulting in a relatively short processing latency. For the Haar transformation, template matching and statistical calculations, 58, 72 and 2 FPGA processing cycles were required respectively, and the corresponding processing time was only 0.58, 0.75, and 0.02 μs, which is almost negligible. Therefore, the total sampling and processing clock cycles of all the sub-processing units were 47 and 168, which attributed to a total sorting latency of ~1.96 ms. If the input signal already has a high SNR, the smoothing module can be bypassed by the users and the sorting latency could further be reduced to ~1.79 ms. These latency results were also confirmed with simulations with Vivado Simulator (Xilinx; San Jose CA) included in Vivado at the gate level.

**Table 1:** Sorting latency of the FPGA based real-time spike soring module.

FPGA sub-processing unit	Clock Cycle		Latency(μs)
	Sampling(24Khz)	Processing(100Mhz)	
Data smoothing	4	2	166.7
NEO peak detection	1	2	41.7
Spike isolation	42	32	1750.3
Haar transformation	NA	58	0.58
Template matching	NA	72	0.72
Firing rate statistics	NA	2	0.02
**Total sorting latency**	**47**	**168**	**1960.0**

For the use of the FPGA resource, the implementation used about 65% of the FPGA slice look-up tables (slice LUTs). Additionally, the amount of slice registers, block memories and bonded input-output blocks (IOBs) were accounted to be 14.5%, 9% and 13.3% of the total available resources respectively.

### Spike sorting accuracy comparing CM and ED using publicly available neural datasets

The sorting accuracy of the FPGA module was also evaluated using publicly available neural recording data [42]. Cluster classification of both CM and ED were evaluated to illustrate the difference between the two techniques. The information gives an indication of which technique is best to use under certain experimental conditions for optimal sorting.

**Fig 5:**
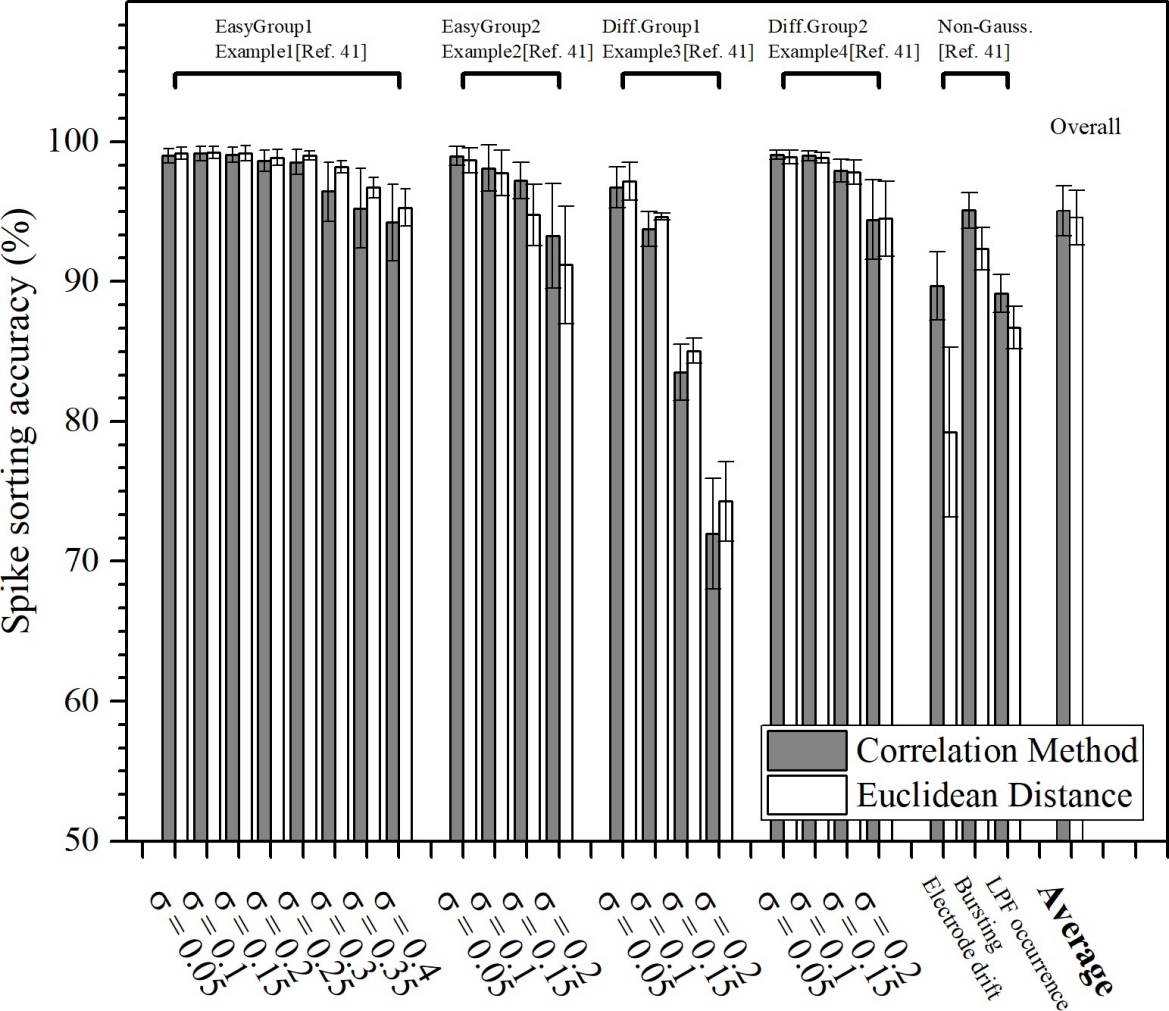
Comparison of the spike sorting accuracies for CM (dark) and ED (white) under various noise contamination conditions. Third party pre-labeled neural spikes were used to estimate the spike sorting accuracy of our system **[42]**. The first 20 sets of spike data were contaminated by Gaussian noise and were separated into four different groups–two groups (EasyGroup1 and EasyGroup2) constructed by spikes that are easily separable and the other two groups (Diff.Group1 and Diff.Group2) constructed by spikes with similar temporal profiles. The final three sets of test data were non-Gaussian noise contamination and were constructed to mimic spike shape changes caused by various physiological conditions (electrical drifting, cell bursting activity, and local field potential occurrence). The results indicate that CM can achieve higher sorting accuracies over ED, especially for neural spikes contaminated with non-Gaussian noise.

Twenty-three sets of artificial neural spike trains, pre-labelled with predetermined classification groups and also contaminated with different types of noise and fluctuations from Ref. [42], were used to test the FPGA module. Among the 23 sets of data, 20 sets of data contained artificial neural spikes contaminated with different degrees of Gaussian noise and the final 3 sets of data were corrupted by non-Gaussian spike height fluctuation. For the 20 sets of data contaminated by Gaussian noise (where *σ* in Fig 5 denotes the standard deviation of the Gaussian noise function), 12 data sets (8 for EasyGroup1 and 4 for EasyGroup2) were constructed by easily separable neural spikes and 8 sets of data (4 for DifficultGroup1 and 4 for DifficultGroup2) were constructed by neural spikes having very similar temporal shapes. The 3 non-Gaussian fluctuation groups were constructed to mimic spike height changes due to various physiological conditions (electrode drifting, cell bursting activity, and local field potentials). Since all data sets were pre-labeled with predetermined cluster groups, it allowed us to compare the sorting results from our system to calculate the sorting accuracy. For each set of data, the first 20 seconds of the data were used as in the training phase to build cluster templates with SPC. The remaining 40 seconds of neural spikes was classified based on template matching, using both matching techniques of CM an ED. The classification results were then compared to the labels of the respective spikes to calculate the sorting accuracy.

Fig 5 illustrates the sorting accuracy of both CM and ED against the 23 data sets. Here sorting accuracy is defined as the ratio between the numbers of spikes that are correctly clustered against the total numbers of spikes. CM showed a slightly better overall sorting accuracy than ED of the 23 datasets examined. Looking more closely at the sorting results, for the 20 sets of data that were contaminated by Gaussian noise (first four groups in Fig 5), ED was actually performing slightly better than CM and the accuracy difference was only about 2%. On the other hand, for the 3 sets of non-Gaussian fluctuation, CM had a significantly better sorting accuracy than that of ED, especially for one set of data that simulated amplitude fluctuation caused by the positional drifting of the metal electrode within the brain during long duration experiments. Under this particular experimental condition, ED had about 80% sorting accuracy while CM could achieve a sorting accuracy as high as 92% (a 12% accuracy enhancement).

**Fig 6:**
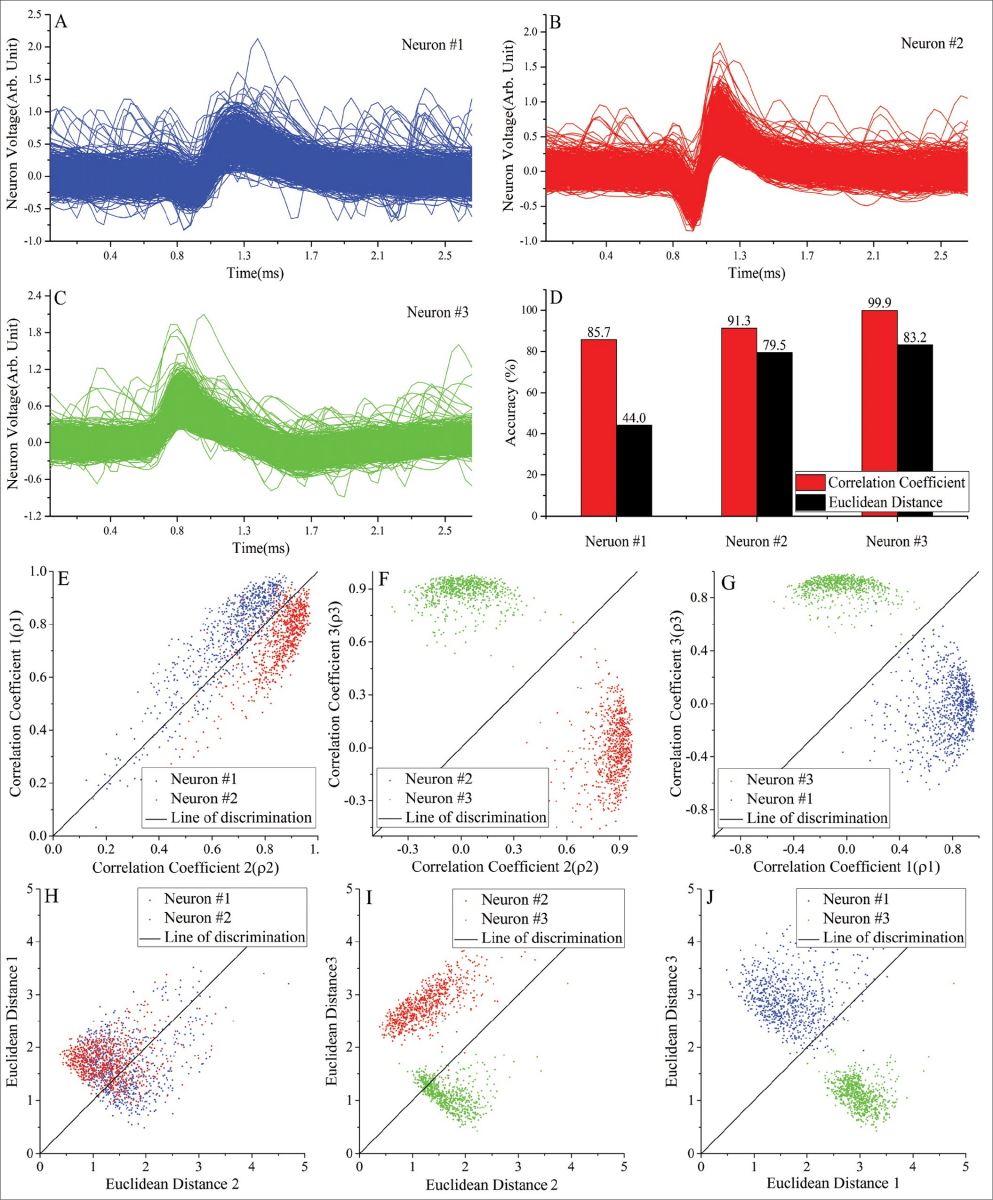
Comparison between CM and ED using neural spikes artificially constructed to simulate electrode drifting. (A) and (B) Temporal profiles of two artificial neural spike clusters with linearly decreased spike amplitudes. (C) Temporal profiles of the third artificial neural spike cluster with linearly increased spike amplitudes. (D) Sorting accuracies of the three neural clusters using CM and ED. (E) to (G) Correlational plots between each of the three correlational coefficients in CM and the figures show clean separation among the cluster groups along the diagonal line. (H) to (J) The diagonal line cannot separate the clusters in ED sorting and significant overlaps can occur.

Additional tests were performed to help understand why CM had a better performance than ED particularly for the experimental scenario of electrode drift. Electrode drift contributes to non-Gaussian noise, therefore a test data set was specifically constructed for this purpose. To construct this test data set, the spikes of three cluster groups contained in one of the public data sets with a medium level of Gaussian noise (C_Drift_Easy2_noise015.mat) were chosen to build this data set. Among these three cluster groups, the amplitudes of the spikes in the first two groups decreased linearly and the amplitude of the spikes in the third group increased linearly to simulate spike height fluctuations due to electrode drifting. The modified spikes were then sent to the FPGA system for testing with both CM and ED template matching. The temporal profiles of the three cluster groups with the artificially varying amplitudes are shown in Fig 6A–6C, Fig 6D shows the sorting accuracy of this test data set using both CM and ED template matching methods. It is evident that ED has inferior performance than CM for all three cluster groups. To better understand these results, the correlation coefficients of any two cluster groups were plotted against one another as shown in Fig 6E and 6F, and the EDs of any two groups were also plotted in Fig 6H–6J. For CM, it is apparent that spike clusters were clearly separated by the diagonal line and the clusters stayed in their own quadrants. In contrast, for ED, the two cluster groups in Fig 6H were intermingled, and the lower cluster groups intrude into the upper quadrant in Fig 6I. These results can be explained by the fact that mathematically the correlation coefficient is much less sensitive to amplitude fluctuation as long as the spike shape is maintained, while ED can change relatively significantly when the amplitude varies. The results also indicate that separating cluster groups in CM can be achieved by using simple diagonal lines to separate the cluster quadrants. In contrast, although several prior studies have used diagonal lines to separate clusters, this is evidently not an optimal technique and more sophisticated comparison algorithms based on cluster boundary segmentation are perhaps required to yield better cluster results for ED [34,36–38,43].

### Spike sorting accuracy evaluated by pre-recorded neural spikes of an anesthetized gerbil

**Fig 7:**
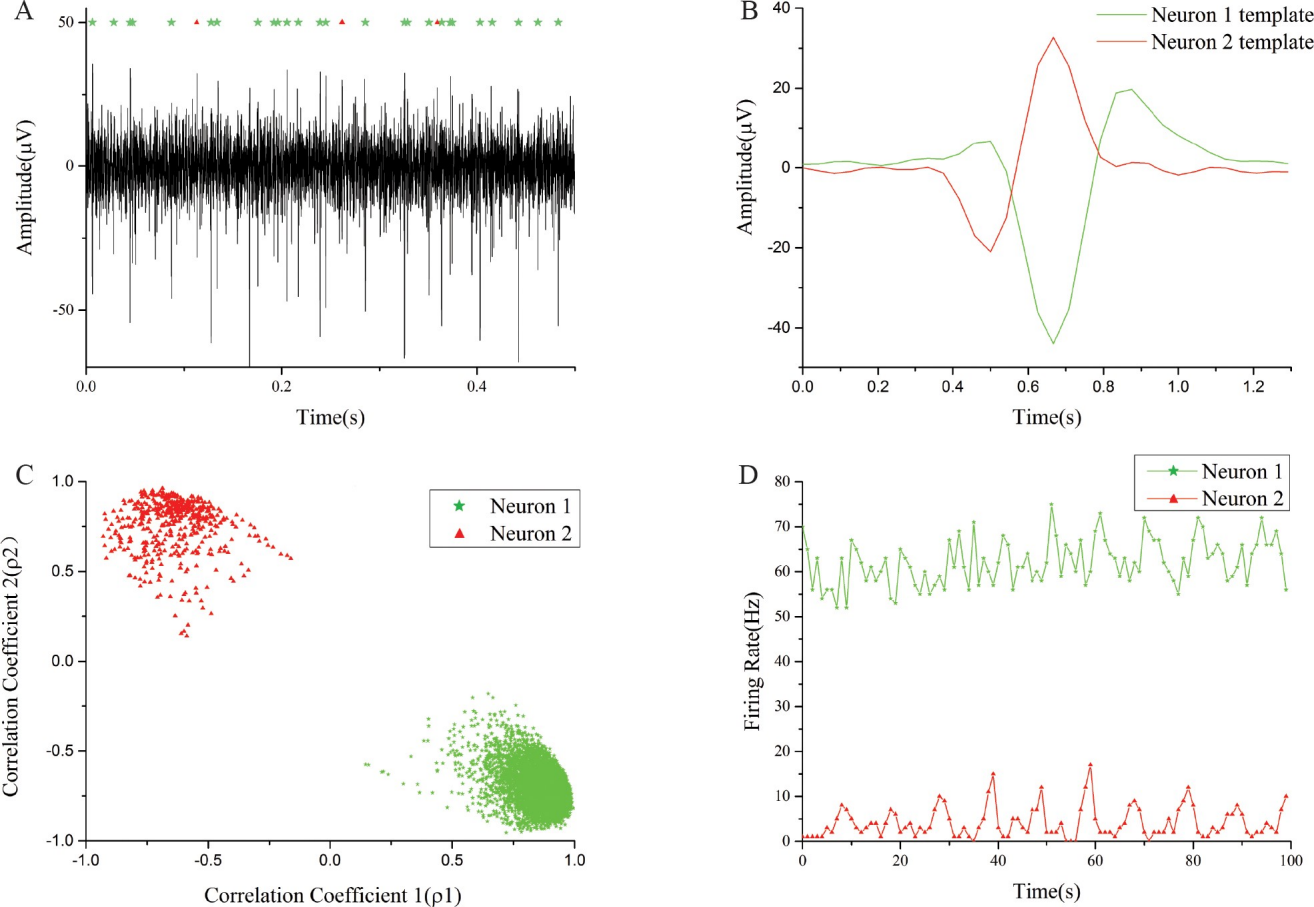
Real-time spike sorting results based on pre-recorded neural spikes from an anesthetized gerbil. (A) 0.5 s of neural voltage trace recorded from the brain stem of an anesthetized gerbil. The green stars and red triangles at the top of the figure indicate the locations of neural spikes of two neurons co-recorded by the same electrode. (B) Temporal profiles of the two cluster templates of the two neurons estimated by SPC. (C) Phase plot of the two cluster groups (green star and red triangle) with each marker representing a neural spike. (D) The firing rates of the two clusters calculated over the 100 seconds of neural data by the FPGA hardware.

Fig 7A shows a portion (0.5 s) of a raw voltage trace recorded from the brain of a Mongolian gerbil (the entire recording was 120 s). The neural voltage contained two distinct types of spikes originating from two close-by neurons that have been identified by the system. Fig 7B shows the averaged temporal profiles of the two cluster templates estimated by the SPC algorithm on the desktop computer, and the template creation used 20 seconds, or 16.6%, of the recorded data. The cluster templates were then transferred to the FPGA for sorting the remaining 100 seconds of data. Fig 7C is the sorted result using CM and represented by a correlational plot of Pearson’s correlation coefficients $\rho_{a}^{i}$ of the two groups. The neural spikes were highly clustered into two groups, indicating that the spikes were well separated. Finally, Fig 7D shows the two time traces of the firing rates of the two cluster groups over the 100 second period. The first neuron maintains a higher firing rate of about 50 to 70 spikes/second, in contrast to the second neuron that fired less than 10 spikes/second for the entire recording.

### Spike sorting agreement evaluated by real-time recordings of an awake behaving mouse

**Fig 8:**
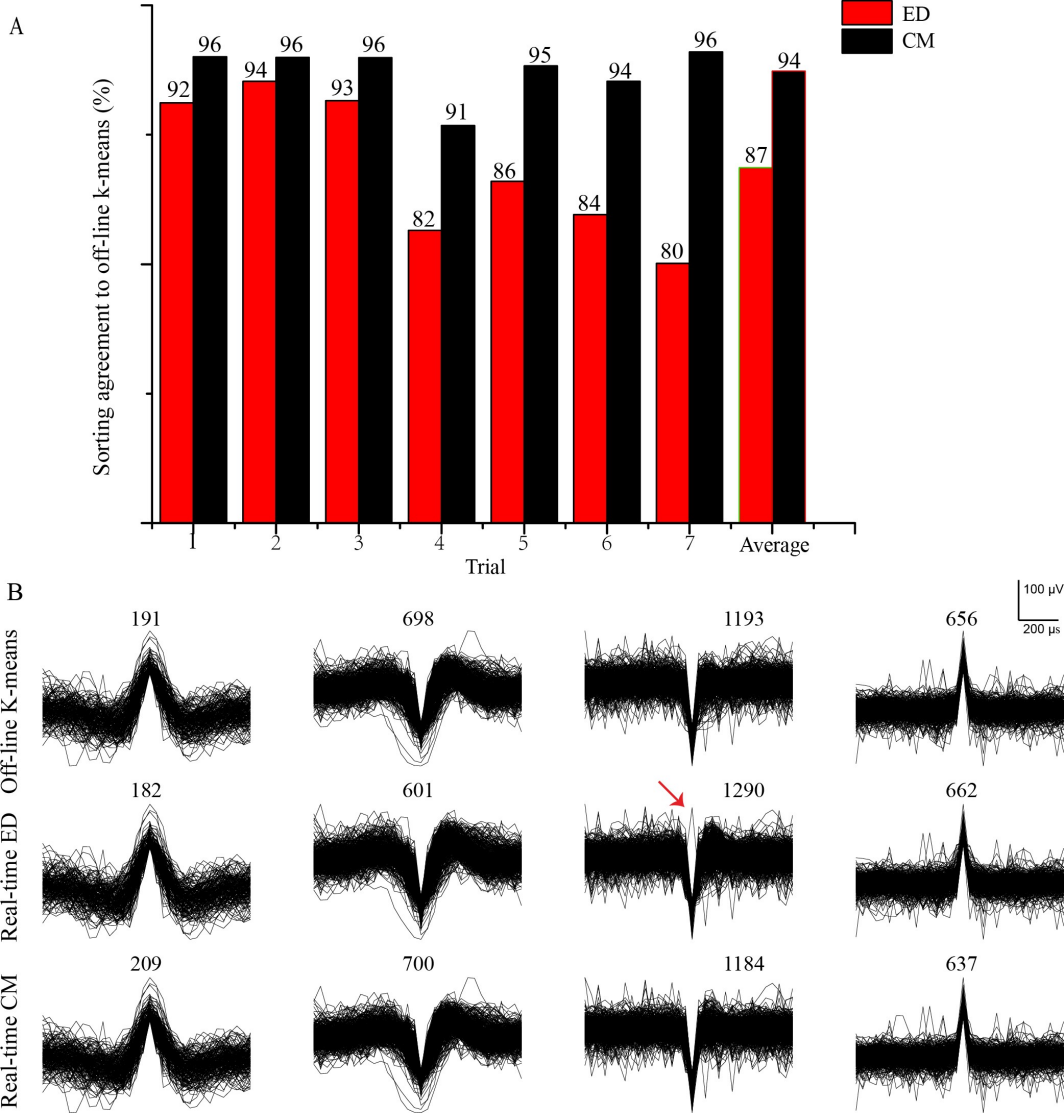
Real-time spike sorting results based on an awake behaving mouse. (A) Real-time spike template matching (ED and CM) compared to off-line k-means classification recorded from the olfactory bulb of an awake behaving mouse. The sorting agreement is higher than 80% for all clusters. (B) Temporal spike profiles (Trial 3) of four clusters sorted by off-line K-means, real-time ED and real-time CM. The numbers at the top of each plot indicate the number of spikes classified to the cluster, and the results indicate similar performance of the three techniques. The red arrow indicates a spike sorting anomaly, likely caused by using vector distances as the sole classification criterion in ED.

Real-time spike sorting was performed on an awake behaving male C57BL/6 mouse and successfully classified the streaming neural spikes using our system, as described in the methods section. During the pre-training period, 4 cluster templates were identified and determined using 2361 of neural spikes with a recorded time of 300 sec. After transferring the template clusters to the FPGA, 2738 of neural spikes were classified in real-time with an experimental time of 300 s. The experiment was repeated 7 trials using the same pre-training templates, with a total experimental time of 2100 s. Once the real-time recording experiments were concluded, a commercial spike sorting software (Offline Sorter, Plexon Inc, Dallas TX) based on k-means clustering was used to sort the same recorded neural spikes off-line. The obtained sorting results were used to compare with those obtained from our real-time template matching methods. In order to calculate the sorting agreements for the two template matching techniques, the off-line sorting results obtained using K-means were considered as the “ground truth” results, and the sorting agreements were calculated by dividing the number of spikes classified to the same group by both the real-time and off-line techniques to the number of spikes classified to the off-line technique only. Fig 8A shows the sorting agreements of ED and CM techniques to the off-line k-means technique. Generally, the two real-time sorting methods have agreements of higher than 80% to the off-line result, and can reach up to 96%. It is also noticeable that CM shows a slightly higher sorting agreement than that of ED (94% vs 87%), and this result is consistent with the results obtained with the publicly available datasets.

Fig 8B shows the temporal spike shapes of four cluster groups sorted by off-line k-means and template matching with both ED and CM modes using our real-time system. As shown in the Fig 8B, both real-time template matching techniques yielded comparable temporal spike shapes and similar total number of sorted spikes for the clusters to those using off-line k-means clustering, which demonstrated the high sorting capability of our real-time system. Comparing the two template matching techniques, ED could occasionally classify a mismatched spike to a cluster group, as indicated by a red arrow of the third cluster for the ED technique. This is likely due to the fact that ED only calculated the vector distances between the templates to the neural spike, and if this spike happened to have the shortest distance template, it was classified to this cluster group even if the shapes were different. In contrast, CM was more sensitive to the general temporal shape of the spike and would reject this spike that had a different temporal spike shape.

### Comparing template matching to other off-line spike sorting algorithms in sorting accuracy and time

**Fig 9:**
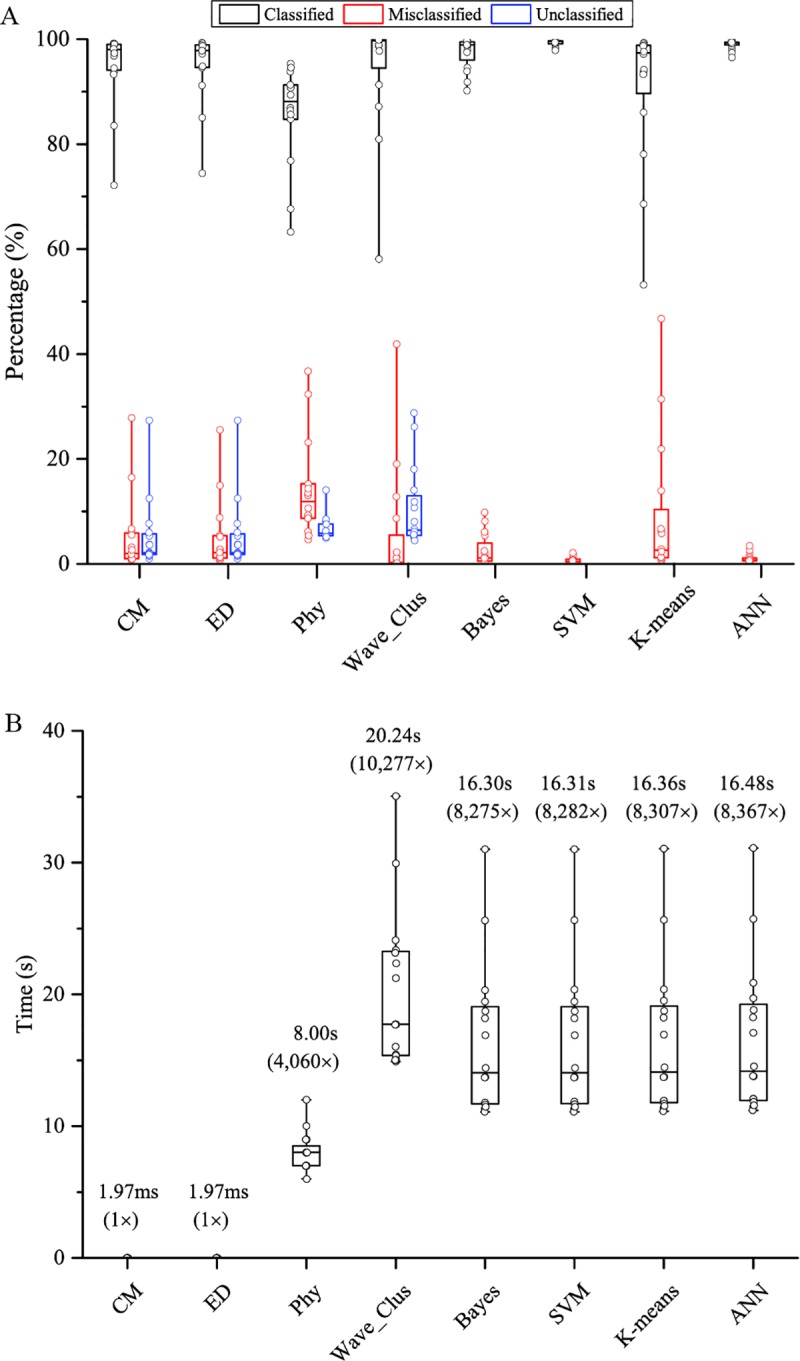
Sorting accuracy and time comparison between CM and ED hardware template matching to other off-line spike sorting algorithms. (A) Percentage ratios of correctly classified (black), misclassified (red) and unclassified (blue) neural spikes comparing several off-line neural spike sorting algorithms to CM and ED hardware template matching using the third party labeled neural spikes [42]. The results indicate that CM and ED achieve comparable sorting accuracies with the other off-line sorting algorithms. (B) Sorting time comparing between hardware CM and ED template matching to other off-line spike sorting algorithms. While CM and ED template matching requires less than 2 ms of sorting time, the off-line spike sorting techniques require sorting time in seconds. Other offline sorting methods used in the comparison: Phy [12,23], Wave_Clus [42], Bayes, SVM (Support Vector Machine), K-means, Artificial Neural Network (ANN) [60].

The sorting accuracy and sorting time between ED and CM template matching techniques to six other off-line spike sorting algorithms was compared in Fig 9. The six off-line spike sorting algorithms used in the comparison are Phy [12,23], Wave_Clus (SPC) [42], Bayes, Support Vector Machine (SVM), K-means and Artificial Neural Network (ANN) [60] Particularly, Phy, which as based on masked EM, is a state-of-the-art spike sorting algorithm for multi-electrode recording, and Wave_Clus, which is based on SPC, is a non-parametric neural spike sorting algorithm for single electrode. Spike sorting with Bayes, SVM, K-means, and ANN techniques are performed with our in-house python software using software routines from the Scikit-learn library. The neural spike data used in the comparison were the same 23 sets of neural spikes used previously in comparing CM and ED techniques [42]. It has been determined that roughly 30 neural spikes are required to generate a good quality cluster template for each neuron, which is in agreement with the findings of Karkare et al [36]. The calculation time for the desktop to determine the templates was measured to be 78.3 seconds, and the times listed in Fig 9 only reflect the processing time of the FPGA hardware to sort a streamed neural spike. The percentages of correctly classified (black), misclassified (red) and unclassified (blue) neural spikes are plotted in Fig 9A. The results indicate that CM and ED have comparable sorting accuracies to all the other spike sorting algorithms. In addition, the sorting times required for the spike sorting techniques to sort the test data are plotted in Fig 9B. EM and CM template matching methods have the shortest sorting time (1.97 ms) among all the methods, and all the other techniques require a sorting time over several seconds.

## Discussion

We developed a real-time rapid neural spike sorting system by matching neural spikes to a group of pre-calculated cluster templates using a hybrid software/hardware approach. This approach significantly reduces the sorting time by matching a neural spike to estimated cluster templates in a single pass using custom digital hardware, eliminating the need for performing sophisticated spike sorting calculations in an iterative manner on a powerful computer. With this approach, the system achieves a maximum sorting rate of 941 spikes/second and a sorting latency of less than 2 ms for a single electrode. This performance is approaching the physiological time characteristics of a neural spike, which has a pulse width of about 1 ms. In addition, the system is flexible by using two kinds of template matching techniques, either through finding the shortest ED or the maximum correlation coefficient among the templates. These two methods are selectable by investigators to fit different experimental sorting needs. Our results indicate that both techniques achieve good sorting accuracies. While ED has a slight edge (about 2% better for both pre-recorded neural spikes and actual animal experiments) over CM for sorting spikes contaminated with Gaussian fluctuation, CM can achieve much better accuracy for pulses that are changing over slow drift of the electrode position in behavioral experiments. These results provide a guideline for choosing the appropriate template matching technique to achieve the best sorting accuracies according to the actual experimental conditions.

Closed-loop neural control is a general technical term referring to interventions of the neural circuit by analyzing responses, either firing rates from electrophysiological recording or behavioral responses of the subject under study, of the neural system in real-time. Closed-loop control provides an exciting opportunity for neuroscience and engineering communities to look into neural systems not only from a passive observational ground, but also from an active control paradigm. To that end, the biochemical technique of optogenetics provides a precise control method that was not available 15 years ago [26,27,29,30,61–63]. Optogenetics allows researchers to stimulate or inhibit a neural system selectively, or simultaneously stimulate and inhibit a neural network. Moreover, with proper biochemical techniques, a specific cell type within the neural target can be specifically or exclusively controlled [29]. Thus combining optogenetics with feedback control, recent experiments have demonstrated the firing rate of a neuron can be controlled for a short period of time [27]. These recent developments provide a good reason for development of rapid spike sorting methods. For instance, the output, such as the firing rate, of rapid spike sorting can be used as the inputs for the closed-loop control routines. In these closed-loop control schemes, the inputs have to be “instantaneous” to reflect the current state of the neural system; thus, rapid sorting and short latency of analyzing neural spikes become important criteria.

When an electrode is inserted into the brain to measure neural voltages, several different kinds of perturbations can contaminate the measured signal. The most common contamination is thermal noise induced by the electrode impedance as well as the intrinsic noise of the amplifier [7,8,63]. Since these noises are stochastic in nature, their noise distributions are typically Gaussian [42]. As shown by our results, ED and CM can handle Gaussian noise almost equally well and the sorting accuracies are good even for low signal-to-noise ratio situations. Besides Gaussian contamination, there are also other types of contamination that are more related to the physiological conditions of the neural system, such as large local field potentials riding on top of weak action potentials [42]. Particularly, for long-term extracellular *in vivo* recordings with freely moving animals, minute movement of the electrodes can change the impedance between the electrode and the neurons, causing the amplitude of the spikes to fluctuate without changing the overall temporal shapes [9,34,42]. Under this scenario, CM has much improved performance over ED since CM is not sensitive to the amplitude change due to the inherent normalization to the signal. In contrast, ED strictly measured the distance between two points in phase space and this distance can change rather substantially as the spike amplitude fluctuates, as demonstrated by occasional spike anomalies to sorted clusters. Another advantage of CM is that cluster decimation is relatively simple through disseminating of the diagonal line, which could further simplify the implementation of CM over ED at the hardware level.

For our real-time template matching system, the maximum sorting rate was measured to be 941 spikes/second with a sorting latency of less than 1.96 ms for a single electrode. Both numbers are not limited by the processing power of the FPGA or the template matching algorithm, but simply reflect the temporal nature of the neural spikes. Physiologically speaking, a neural spike has a pulse width of ~1 to 2 ms and a neuron typically cannot fire more than several hundred of spikes per second, both of which are limited by the molecular dynamics of the Na and K ion channels [63]. Thus, using an analog-to-digital converter with higher sampling frequency will not help to improve the sorting rate simply because the sorting cannot occur without the entire spike being sampled. For this reason, we believe that our current system is approaching the limits of how fast a system can be in sorting neural spikes, at least in the case of single channel sorting, unless non-causal techniques are developed to predict spike profiles.

We compared the sorting accuracies and the sorting speed of our system using template matching techniques to other off-line spike sorting algorithms. Based on the results, the sorting speed of hardware template matching is three orders-of-magnitude faster than those of the other methods. This is due to the fact that template matching can sort a neural spike immediately once it is measured, while the other methods take an iterative approach to examine all the neural spikes at the same time in order to seek the best possible match. On the other hand, the comparisons also indicate that the sorting accuracies obtained with template matching techniques are comparable to those of other sorting methods, making template matching highly attractive for real-time spike sorting applications. Recently, our team published a new spike sorting algorithm, named Enhanced Growing Neural Gas (EGNG), that utilize this single pass concept to learn neural spike cluster distributions on the fly and immediately classify neural spikes in real-time [64]. Not only is this new algorithm fast and implementable using digital electronic technology with limited computational resources, it is also highly adaptable to changes in electrophysiological environments. We plan to combine the template matching techniques with the EGNG algorithm to remove the need of a desktop computer to create a completely tether-free and portable neural spike sorting IC to demonstrate closed-loop neural control with animal models in the near future.

The current system is limited to processing neural spikes recorded from a single electrode. The system, however, can be extended in the future to process neural spikes recording from multiple electrodes. It is worth mentioning that data sampling and smoothing, peak detection and spike isolation (1.75+0.1667+0.0417 = 1.958 ms) contribute to 99.9% of the sorting latency (1.958/1.9660 = 99.9%). This is due to the fact that a biological neural spike has a pulse width between 1 to 2 ms and template matching cannot be performed until the neural spike was sampled. Once the neural spike was measured and isolated, feature extraction and template matching were extremely fast and only took a very short time to process (0.58 + 0.72 + 0.02 = 1.32 μs or 0.00132 ms). Therefore, a maximum matching speed of ~0.75 million spikes/second can be achieved for the template matching alone with our hardware. Therefore, to create a system to template match neural spikes measured from multiple electrodes, neural amplifiers integrated with the hardware peak detection and spike isolation units can be designed for each recording electrode. A multiplexer with a FIFO memory unit can be used to arrange and store all the isolated neural spikes measured from the electrodes, and the template unit can also be modified to match all the isolated neural spikes while preserving the originating electrode site indexes.

Additional hardware units can also be developed to handle temporally overlapping spikes, as well as the same neural spike picked up by multiple closely spaced adjacent electrodes. Recent off-line spike sorting algorithms used similar methods to sort temporally overlapping neural spikes by matching the temporally overlapping neural spikes to the single unit neural spike templates determined during the first processing phase of the algorithm [20]. Using the same idea, it is possible to design a hardware module to match temporally overlapping neural spikes to multiple single-unit neural templates in real-time. In addition, closely spaced electrodes that pick up duplicate neural spikes emitting from the same neuron can be correctly processed by hardware using the unique signal properties these duplicate spikes contained. The electrode closest to the emitting neuron picks up the largest signal amplitude while the adjacent electrode has a reduced amplitude inversely proportional to the distance between the electrode and the emitting neuron [65,66]. The arrival time differences between these neural spikes are less than 1 ms and the neural spikes can be approximately considered arriving at the electrodes at the same time [20]. Therefore, a hardware unit can be designed to compare neural spikes measured from adjacent electrodes and arriving to the electrodes at approximately the same time to determine the neural spikes with the largest amplitude while rejecting the rest to avoid counting duplications. We are currently working on the next version of our system that incorporates these hardware units to allow multiple electrodes and handle overlapping spikes.

We have tabulated some of the features of other recent real-time spike sorting systems which were implemented with either ASIC or FPGA technologies and compared them to our system in Table 2. All other systems have also implemented some form of spike detection, feature extraction, and only ED as their template matching method [36–38,67]. However, our system took advantage of these previous developments and demonstrated high sorting accuracies, tested with public neural data sets, pre-recorded neural spikes and real-time spike sorting of an awake behaving mouse. Our system is also capable of using either ED or CM template matching techniques to obtain optimal sorting results based on the neural physiological conditions during the recording. In addition, our system also implemented a real-time statistical module to calculate the instantaneous firing rates. The real-time statistical module may open up new opportunities in the future for downstream neural data analysis based on instantaneous firing rates, such as in closed-loop neural controls for neurophysiological disease managements.

**Table 2:** Performance summary and comparison with others work.

	[36]	[37]]	[38]	[67]	Methods in this paper
**Spike detection**	Y	Y	Y	Y	**Y**
**Feature extraction**	N	N	N	N	**Y**
**Template matching technique**	ED	ED	ED	ED	**CM / ED**
**No. of channel**	16	1	128	128	**1**
**Process (nm)**	65	FPGA	FPGA	65	**FPGA-28nm**
**Sorting Latency (ms)**	N/A	11	N/A	N/A	**1.96**
**Max. spike rate (spikes/sec)**	70	88	N/A	N/A	**941**
**Real-time statistics**	N	N	N	N	**Y**
**Power**	75uw	N/A	N/A	22.4uw	**0.46w**
**In vivo verification**	N	N	N	N	**Y**

## Conclusion

We have developed a real-time neural spike sorting with low sorting latency and high sorting throughput using template matching techniques and compared two template matching methods (ED and CM) for their optimal uses in real-time neural spike sorting. The system consists of a desktop computer (software) to generate cluster templates and an FPGA (hardware) to match subsequent incoming spikes to the templates in real-time. The two template matching methods are user selectable for best sorting results. Both ED and CM are good for sorting spikes contaminated by regular Gaussian noise typically introduced by instrumentation and CM is best for other atypical noise, such as positional drift of electrodes. The system was characterized by publicly available neural spike datasets, pre-recorded neural spikes from an anesthetized gerbil. The sorting performance and accuracy of the system was further evaluated by neural spikes recorded from an awake behaving mouse in real-time, and compared to other neural spike sorting algorithms, confirming the system is readily usable in real-time neural spike analysis applications. The maximum spike sorting rate is 941 pulses/second with a short sorting latency of less than 2 ms. These characteristic parameters are only limited by the intrinsic pulse width of a neural spike (1–2 ms), but not by the calculating performance of the FPGA and the efficiencies of the template matching algorithms.

## Supporting information

S1 Text Hardware implementation details of spike detection module and Haar transformation module. The supplementary file describes hardware implementation details of peak detection, spike alignment on spike detection module, also including Haar transformation module for extracting the features of detected spikes.
(DOCX)

S1 Video Demonstrating real-time spike sorting system processing on an awake behaving mouse. (MOV)

S1 Data Two real neural recording datasets are used in this paper. One is recorded from an anesthetized gerbil, the other is from an awake behaving mouse on in-vivo experiment.
(ZIP)

S1 Code The functions of the custom-written python program include two parts: 1) performing spike sorting, and 2) extracting templates based on spike sorting results. There is a readme file to indicate how to use this program.
(ZIP)
