# Supplementary material for: Low-latency single channel real-time neural spike sorting system based on template matching
Source: PLoS One. 2019 Nov 22;14(11):e0225138. doi: 10.1371/journal.pone.0225138 (PMC6874356; doi:10.1371/journal.pone.0225138)
Supplement: S1 Text — The supplementary file describes hardware implementation details of peak detection, spike alignment on spike detection module, also including Haar transformation module for extracting the features of detected spikes. (DOCX) [file pone.0225138.s001.docx]

**Hardware implementation details of spike detection and Haar transformation**

**1. Peak detection and spike alignment in spike detection module**

Fig 1A illustrates the peak detection and spike alignment modules implemented in the FPGA before template matching. The purpose of these two modules is to isolate neural spikes onto a spike array with 32 data samples for each spike from the continuous digitized neural voltage x[n]. The continuous digitized neural voltage x[n], which were contaminated with environmental high frequency noise, was first processed by a data smoothing module before the spike isolation to remove this high frequency noise. The smoothing module, however, can add a short processing time additional to the overall sorting latency. Therefore, for some applications requiring rapid analysis such as dynamic closed-loop neural controls, the smoothing module can be optionally bypassed to save processing time, especially when the SNR of the recorded neural spikes is relatively high.

After the neural spikes x[n] were smoothened, the peak detection module detected the presence of neural spikes from x[n] and identified their peak positions. Two peak detection methods - amplitude threshold detection and nonlinear energy operator (NEO) – were implemented in the module to identify neural spike peaks and these two methods were selectable by users. Based on the determined peak position, the spike isolation module separated the neural spikes from the continuous neural spike train x[n] and sequentially transferred the isolated data onto a First-In First-Out (FIFO) spike windows with 64 sample space.The purpose of storing the neural spike data onto the FIFO was to allow the peaks of the isolated neural spikes to be aligned to an off-centered position, as shown in Fig 1B-1C This peak shifting feature avoided the isolation procedure to cut away off-centered features of the neural spikes to allow better classification downstream. The center array position was adjustable by users and with the peak index counter and timing control, the FIFO could shift the center position and fit the neural spike encompassing the off-centered feature onto a 32-samples array for downstream classification.


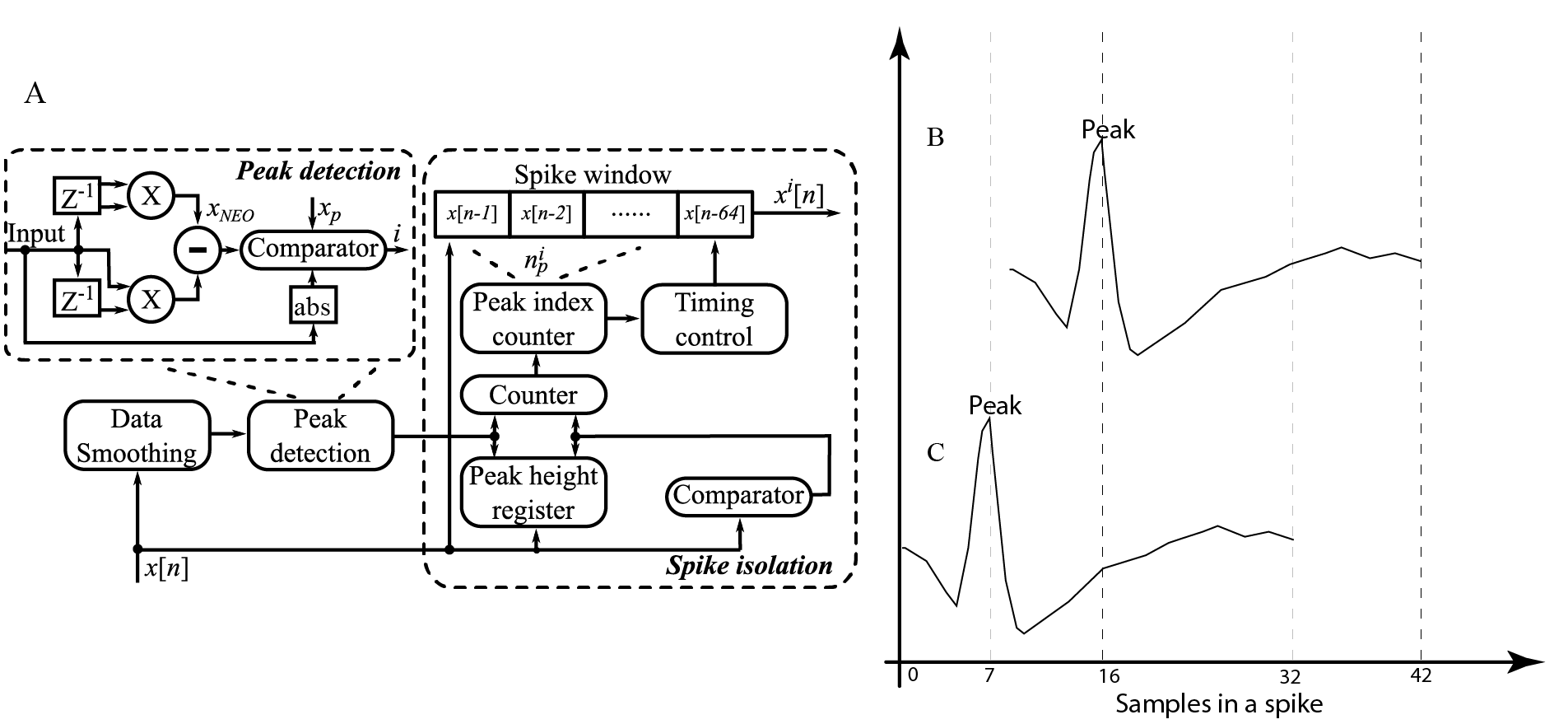


Fig 1. (A) The block diagram of the spike detection and peak alignment module for downstream classification based on template matching. Neural spikes were isoloated onto a 32-samples array of individual neural spikes. (B) Illustration of a neural spikes with off-centered temporal feature that could not be fit into a 32 sample space. If the spike were not shifted before isolation, the off-centered temporal feature would have been discarded, hampering the classifcation accuracy. (C) The peak alighnment module allows a user specific shift to allow the off-centered temporal feature to be included within the 32 sample space.

**2. Haar wavelet transformation module**

After neural spike isolation, a hardware module for Haar wavelet transform was implemented to extract the spike characteristics. The Harr wavelet transformation module was constructed with four processing levels with similar hardware architecture, as shown in Fig 2A. For a neural spike $x^{i}[n]$, the wavelet coefficient ${\overset{\to}{w}}^{i}=\{a_{4}^{i},d_{4}^{i},d_{3}^{i},d_{2}^{i},d_{1}^{i}\}$ can be calculated using the following equations in 4 sequential levels.

Level 1 $d_{1}^{i}\left[ n \right]=\frac{x^{i}\left[ 2n \right]-x^{i}\left[ 2n+1 \right]}{\sqrt{2}}$

$a_{1}^{i}\left[ n \right]=\frac{x^{i}\left[ 2n \right]+x^{i}\left[ 2n+1 \right]}{\sqrt{2}}$ for $n=0\ldots15$

Level 2 $d_{2}^{i}\left[ n \right]=\frac{a_{1}^{i}\left[ 2n \right]-a_{1}^{i}\left[ 2n+1 \right]}{\sqrt{2}}$

$a_{2}^{i}\left[ n \right]=\frac{a_{1}^{i}\left[ 2n \right]+a_{1}^{i}\left[ 2n+1 \right]}{\sqrt{2}}$ for $n=0\ldots7$

Level 3 $d_{3}^{i}\left[ n \right]=\frac{a_{2}^{i}\left[ 2n \right]-a_{2}^{i}\left[ 2n+1 \right]}{\sqrt{2}}$

$a_{3}^{i}\left[ n \right]=\frac{a_{2}^{i}\left[ 2n \right]+a_{2}^{i}\left[ 2n+1 \right]}{\sqrt{2}}$ for $n=0\ldots3$

Level 4 $d_{4}^{i}\left[ n \right]=\frac{a_{3}^{i}\left[ 2n \right]-a_{3}^{i}\left[ 2n+1 \right]}{\sqrt{2}}$

$a_{4}^{i}\left[ n \right]=\frac{a_{3}^{i}\left[ 2n \right]+a_{3}^{i}\left[ 2n+1 \right]}{\sqrt{2}}$ for $n=0\ldots1$

Since the equations of the 4 levels are similar, the levels were implemented similarly with adders and subtractors, as shown in Fig 2B. The$\sqrt{2}$ division was approximated by right shifting the bits based on the following equations for efficient hardware implementation. In our design, not all the 32 output wavelet features were used for template matching, through Kolmogorov-Smirnov (KS) test, we select the coefficients which have biggest deviation from normal distribution as representative of spikes and send these selected coefficient into the hardware classifier to conduct spike sorting, and based on our experience 20 or fewer coefficients were typically adequate to differentiate the neural spikes correctly.

$$\frac{x[n]}{\sqrt{2}}=\left( 0.70710\ldots\right)x\left[ n \right]\approx\left( 2^{-1}+2^{-3}+2^{-4}+2^{-6}+2^{-8} \right)x\left[ n \right]=(0.70703\ldots)x\left[ n \right]$$

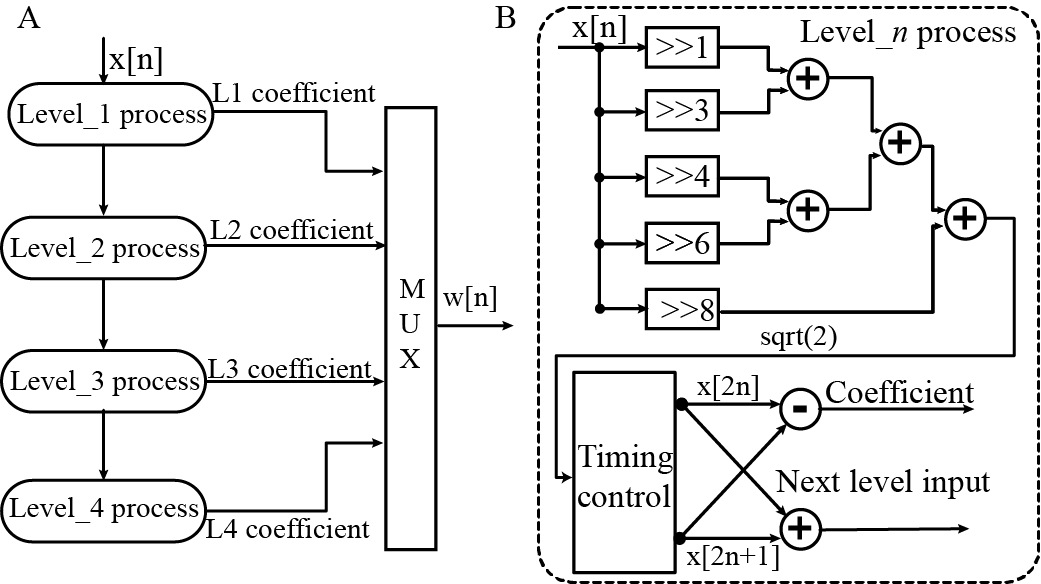


**Fig 2.** (A) The hardware implementation of the Haar wavelet transformation, isolated neural spike x[n] was transformed to generate the Haar coefficients w[n]. There were four levels of processing to generate all the Haar coefficients, and all the four levels of processing had the same hardware structure. (B) A ‘level_*n* process’ structure is illustrated with details.

**3. Supplementary experimental video**

This supplementary video shows a demonstration of our system in which electrophysiological data was recorded for real-time spike sorting from an awake behaving mouse using the template matching technique. The sorted neural spikes can be seen displayed on the control software user interface in real-time. The video also shows the FPGA hardware and the rodent freely moving on a track ball during the neural recording.
